# Risk factors for severe outcomes of respiratory syncytial virus infection in children: a nationwide cohort study in Sweden

**DOI:** 10.1016/j.lanepe.2025.101447

**Published:** 2025-09-09

**Authors:** Giulia Dallagiacoma, Cecilia Lundholm, Awad I. Smew, Emma Caffrey Osvald, Pekka Vartiainen, Santtu Heinonen, Tobias Alfvén, Catarina Almqvist, Samuel Rhedin

**Affiliations:** a Department of Medical Epidemiology and Biostatistics, Karolinska Institutet, Stockholm, Sweden; b Department of Perioperative Medicine and Intensive Care, Karolinska University Hospital Solna, Stockholm, Sweden; c Karolinska University Hospital, Astrid Lindgren Children's Hospital, Stockholm, Sweden; d Institute for Molecular Medicine Finland (FIMM), University of Helsinki, HiLIFE, Helsinki, Finland; e Department of Pediatrics, University of Helsinki and Helsinki University Hospital, Helsinki, Finland; f Centre for Fertility and Health, Norwegian Institute of Public Health, Oslo, Norway; g Finnish Vaccine Research (FVR), Tampere, Finland; h Department of Global Public Health, Karolinska Institutet, Stockholm, Sweden; i Sachs' Children and Youth Hospital, Stockholm, Sweden

**Keywords:** RSV, Respiratory syncytial virus, Bronchiolitis, Risk factors, Cohort study, Pediatric infectious diseases, Epidemiology

## Abstract

**Background:**

While risk factors for respiratory syncytial virus (RSV) hospitalization are well established, few studies have assessed severe disease outcomes. We investigated risk factors for RSV-associated severe disease outcomes in children 0–18 years.

**Methods:**

A register-based cohort study including all children born in Sweden between 2001 and 2022 was performed. Data on RSV related ICD-10 diagnoses, sociodemographic factors and comorbidities were retrieved from national registers. The outcomes were RSV-associated death, Intensive Care Unit (ICU) admission, and prolonged hospitalization (≥7 days). Adjusted hazard ratios (aHR) and 95% confidence intervals (CI) were calculated using multivariable Cox regression both in the full cohort and in the subpopulation of children with an RSV diagnosis.

**Findings:**

Among 2,354,302 children, 38,919 (1·7%) had an RSV diagnosis. Of these, 4621 (11·9%) had severe disease outcomes. The median age of children admitted to ICU were 1·9 months and 500 (41·3%) had an underlying comorbidity. Birth in winter (HR 2·96, 95% CI: 2·53–3·46), small for gestational age (aHR 3·91, 95% CI: 3·08–4·97), multiple birth (aHR 3·43, 95% CI: 2·80–4·21), having siblings 0–3 years (aHR 2·92, 95% CI: 2·57–3·31), and comorbidities (aHRs > 4) were the factors most strongly associated with ICU admission or death in the full cohort. Similar, but attenuated, associations were seen among children with an RSV diagnosis. Comorbidities were less common in severe cases under 3 months of age than in older children (40·3% vs 71·6%, *p* < 0·0001).

**Interpretation:**

Severe RSV cases often affect healthy, full-term infants under 3 months, beyond those with severe comorbidities. Risk factors such as small for gestational age, multiple births, and young siblings are not currently included in RSV immunization strategies, but should be considered to better target vulnerable infants.

**Funding:**

Financial support was provided by KID funding from 10.13039/501100004047Karolinska Institutet, the 10.13039/501100004359Swedish Research Council, the 10.13039/501100003793Swedish Heart Lung Foundation, the 10.13039/501100010234Swedish Asthma and Allergy Association Research Fund, grants from Region Stockholm, the Strategic Research Program in Epidemiology at 10.13039/501100004047Karolinska Institutet, The Society for Child Welfare, 10.13039/100007435Åke Wiberg Foundation, 10.13039/501100010664Martin Rind Foundation, Karolinska Institutet Research Foundation Grants, and the Foundation Freemason Children's Home in Stockholm.

**Research in context**

**Evidence before this study**

We searched PubMed for articles published until June 5, 2024, using the search terms “severe”, “bronchiolitis”, “RSV”, “death”, “intensive care unit”, “hospitalization”, and “risk” as well as their synonyms in the title or abstract. We also included the following MeSH terms: “Respiratory Syncytial Virus Infections”, “Bronchiolitis, Viral”, “Intensive Care Units, Neonatal”, “Intensive Care Units, Pediatric”, “Infant Death”, and “Child Mortality”. Our search identified a limited number of studies that primarily focused on hospitalization in RSV-infected children, with fewer studies investigating severe adverse outcomes such as death, ICU admission and prolonged hospitalization. Additionally, many of the available studies were limited by small sample size, single-center designs, or short follow-up periods.

**Added value of this study**

Unlike most studies focused on hospitalization, ours addresses a key gap by examining the most severe RSV outcomes. Leveraging a large cohort of over 2·3 million children, we were able to investigate rare outcomes and comorbidities that are often difficult to study due to limited statistical power in smaller cohorts. Our study can therefore provide unique, generalizable information on the risk factors for severe RSV disease in a high-income setting over an extended timeframe.

**Implications of all the available evidence**

Our findings emphasize the need for public health interventions targeting all children, including full-term healthy infants, rather than focusing solely on those with underlying conditions. This strengthens the evidence supporting universal RSV immunization in children. In settings where universal immunization is not yet feasible, our study provides crucial evidence to guide the prioritization of high-risk groups, not only based on underlying conditions, but on age and other risk factors including being born small for gestational age, multiple birth, and the presence of siblings aged 0–3 years. Additionally, our findings highlight the importance of caution with all children in clinical practice, as severe RSV outcomes can occur even in healthy infants.

## Introduction

Respiratory syncytial virus (RSV) is the leading cause of acute lower respiratory tract infections (LRTIs) in young children, with the highest burden of disease in low- and middle-income countries. In 2019 there were globally 33 million cases of RSV in children younger than 5 years, leading to 3·6 million hospitalizations and over 100,000 RSV-related deaths.^1^ In Europe, RSV accounts for around 245,000 hospital admissions annually among children under 5 years of age, with the majority (75%) occurring in infants younger than 1 year.^2^ In Sweden, approximately 2000 children are hospitalized due to RSV each year, corresponding to about 9 per 1000 children, with nearly 10% requiring intensive care (ICU) admission.^3^

The risk factors for RSV hospitalization have been extensively studied, but less is known about the specific factors contributing to severe disease outcomes such as death, ICU admission, or prolonged hospitalization, which are the most critical events to prevent but usually hard to study due to limited statistical power. Young age, prematurity, underlying chronic diseases (particularly congenital heart defects and chronic lung diseases), and having older siblings in preschool age are recognized as key risk factors for RSV infection and hospitalization.^4^^,^^5^ However, the relationship between some of these risk factors and hospitalization remains unclear, as infants with comorbidities may sometimes be admitted for overnight observation at a lower threshold due to their increased fragility. In contrast, the predictors of severe disease outcomes, which are less influenced by subjective decision-making, have primarily been examined in smaller patient cohorts. Previous studies suggest that factors such as prematurity, age <6 months, low birth weight, comorbidities, and certain clinical parameters, such as hypoxia at admission, are associated with an increased risk of ICU admission.^6^, ^7^, ^8^, ^9^

Recent advancements in immunoprophylaxis to prevent RSV in children, either through long-acting monoclonal antibodies or maternal vaccination, have offered novel opportunities to prevent RSV and have shown a significant reduction (around 80%) in RSV hospitalizations and severe disease outcomes.^10^^,^^11^ Given the high effectiveness of these new strategies, it is crucial to identify the populations most at risk for severe outcomes in order to shape immunization guidelines and guide public health policies.^12^, ^13^, ^14^ In Sweden, RSV prophylaxis was previously limited to a small number of high-risk children receiving palivizumab. New national guidelines issued in April 2025, planned to be implemented in the RSV season 2025–2026, recommend nirsevimab for all infants under 3 months of age during the RSV season, as well as for children under 12 months with risk factors for severe RSV infection. Children up to 24 months who remain at elevated risk are also eligible during their second RSV season.^15^ Given these recent changes, understanding specific risk factors for severe disease remains essential to guide future developments and optimize resource allocation.

The primary aim of this study was to identify sociodemographic risk factors and comorbidities associated with severe outcomes of RSV infection among children in a high-income setting. Specifically, we aimed to assess risk factors for: I) RSV-associated ICU admission or death, and II) RSV-associated prolonged hospitalization (≥7 days), both at the population level and within the subgroup of children diagnosed with RSV infection. As a secondary aim we investigated the proportion of underlying severe comorbidities in different age groups of children with RSV-related severe outcomes.

## Methods

### Study design and population

This is a register-based nationwide cohort study. All Swedish residents have a unique identifier, which allows unambiguous linkage across all registers. For this study, the Total Population Register (TPR) was linked with: I) the National Patient Register (NPR), that contains International Classification of Diseases - 10th Revision (ICD-10) codes for all hospital visits and the majority of visits to specialist outpatient clinics; II) the Medical Birth Register (MBR) that contains data on perinatal parameters; III) the Swedish Prescribed Drug Register (SPDR) that contains data on all dispensed medications since July 2005; IV) the National Cause of Death Register, which provides the causes of death based on medical death certificates; V) the Longitudinal Integrated Database for Labor Market Studies (LISA), containing data on households and education; VI) the Multi-Generation Register (MGR), containing data on the parents of index subjects; VII) the Swedish Intensive Care Registry (SIR), which provides data on ICU and Pediatric Intensive Care Unit (PICU) hospitalizations (PICU since 2008). Descriptions and references for each of the national health registers used in this study are provided in the Supplementary Material. All children born in Sweden between January 1st, 2001 and December 31st, 2022 were included in our study. Children were excluded if they died within the first 7 days of life (due to the rarity of RSV infection and to reduce bias from early neonatal fragility) or if it was not possible to link them to the maternal identifier. To discriminate between factors that increase the likelihood of medically attended RSV infection at the population level and factors associated with increased risk for a severe disease course once infected, we performed a subgroup analysis restricted to children with a diagnosis of RSV infection in the NPR. Supplementary Figure S1 shows the data availability from each register across the study period.

### Study variables

The main outcomes were: (1) RSV-associated ICU/PICU admission or death, identified through the Swedish Intensive Care Registry and Cause of Death Register; and (2) RSV-associated prolonged hospitalization, defined as a hospital stay of ≥7 days, based on data retrieved from the NPR. RSV association was determined by a recorded diagnosis of RSV infection (ICD-10 codes J12·1, J20·5, or J21·0). RSV-related deaths were those in which one of these ICD-10 codes was listed as the underlying or contributing cause of death in the Cause of Death Register, which is based on death certificates completed by licensed health practitioners. Both PICU and ICU admissions were included, as not all hospitals have a PICU, and children may therefore be admitted to a regular ICU in these settings. Data on ICU admissions were available until December 2021, data on deaths until December 2023 while data on prolonged hospitalizations were available up to March 2024. Children were followed from the 8th day of life for at least 15 months and up to 18 years, depending on the outcome and data availability in each register. In the case of multiple hospitalizations or ICU admissions, only the first event was considered, as repeated events were rare or occurred in close succession, reflecting a single illness episode rather than distinct, independent events.

Sociodemographic characteristics and comorbidities were assessed as possible risk factors and confounders, based on the existing literature on risk factors for RSV hospitalization and on the current Swedish recommendations for RSV prophylaxis.^4^, ^5^, ^6^^,^^8^^,^^9^^,^^15^, ^16^, ^17^ Data on sex (sex assigned at birth, male/female), season of birth (Spring: March–May; Summer: June–August; Autumn: September–November; Winter: December–February), birthweight (continuous, in grams), small for gestational age (SGA, defined according to Maršál et al.^18^; yes/no), early pregnancy Body Mass Index (BMI) (classified as underweight: <18·5 kg/m^2^; normal weight: 18·5–24·9 kg/m^2^; overweight: 25–29·9 kg/m^2^; obese: ≥30 kg/m^2^), multiple birth (yes/no), gestational age (recorded as completed weeks of gestation), maternal age (classified as <25 years, 25–29 years, 30–34 years, and ≥35 years, based on previous findings on child morbidity in relation to maternal age^19^) and maternal smoking during early pregnancy (yes/no; recorded at the first antenatal visit) were retrieved from the MBR. Prematurity, defined as gestational age less than 37 weeks, was further classified following the World Health Organization (WHO) classification into three subgroups: extremely preterm (less than 28 weeks), very preterm (28 to less than 32 weeks) and moderate to late preterm (32–37 weeks).^20^

Data on parental country of birth (both parents born in Sweden; one parent born in Sweden and one abroad; both parents born abroad), parental educational level (classified as highest educational level attained by any of the parents at the time of birth: primary school (0–9 years), secondary school (10–12 years) or university (>12 years)), emigration (yes/no), presence of children living in the same household (yes/no; further categorized as 0–3 and 4–6 years old) were retrieved from LISA. Data on siblings hospitalized for viral LRTI before 4 years of age (yes/no) were retrieved by linking information in the MGR and NPR (ICD-10 codes J20–J21). Family history of asthma (yes/no) was defined using a previously validated algorithm (details are available in Supplementary Material), based on asthma diagnoses recorded in the NPR and asthma medication prescriptions from the SPDR, for the parents or older siblings of the index children.^21^

The following comorbidities (all classified as yes/no) were investigated as possible risk factors for severe adverse outcomes: congenital heart disease (CHD, further classified as severe CHD or uncomplicated atrial/ventricular septal defects), chronic lung disease, neonatal respiratory conditions, trisomy 21, cerebral palsy, esophageal malformations and life-limiting conditions (LLC). These comorbidities were defined based on records of specific ICD-10 codes in the NPR (Supplementary Table S1). LLC was defined as conditions associated with a significantly shorter life expectancy and for which there is no available curative treatment, as previously defined by Fraser et al. (Supplementary Table S2).^7^ The final dataset contains minimal missingness overall. The variables with the highest percentage of missing values are parental education, presence of siblings aged 0–3 years and 4–6 years (each with 6% missing values), followed by exposure to smoking during pregnancy (4%) and small for gestational age (3%). All other variables had either no missing data or less than 0·1% missingness. This resulted in a maximum missingness of 13% in the analysis for the variable “small for gestational age”, while all other analyses had missingness <7%.

### Statistical analyses

Hazard ratios (HRs) and 95% confidence intervals (CIs) for death or ICU admission (grouped as a single outcome due to limited statistical power) and for prolonged hospitalization were estimated for each risk factor using multivariable Cox regression analysis, adjusting for potential confounders and using age as the time scale (using the built-in stcox command). Children were followed from the 8th day of life until they experienced any of the outcomes or were censored. Study subjects were censored in the analyses when they turned 18 years, emigrated, or died. For ICU admission, children entered the risk set starting from January 1st, 2008, the date from which ICU data were available. In a sub analysis, only the children with a record of RSV diagnosis were investigated. To account for non-proportional hazards, we used a flexible parametric survival model using the user-written stpm3 command (modeled on the log-hazards scale with 3 degrees of freedom for both baseline hazard and time-varying effect) to test interactions with age for the variables that demonstrated a significant change in HR by age (namely, prematurity and season of birth).^22^ For all other risk factors, the proportional hazards assumption held, indicating that HR were constant over age and therefore applicable beyond the first RSV season.

Potential confounders were defined separately for each studied risk factor by creating directed acyclic graphs based on the literature (Supplementary Table S3 and Supplementary Figure S2).^4^, ^5^, ^6^^,^^8^^,^^9^^,^^17^ For sex, season of birth and parental birth country, no confounders were identified, therefore these risk factors were only assessed in unadjusted analyses. Esophageal malformations and cerebral palsy were grouped with LLC into an “Other severe comorbidities” group for the analysis due to statistical power considerations.

Additionally, we investigated the proportion of underlying comorbidities in children with a severe RSV-related outcome, specifically: prematurity, CHD (both severe CHD and uncomplicated atrial/ventricular septal defects), chronic lung disease, neonatal respiratory conditions of the term child, trisomy 21, LLC, cerebral palsy, esophageal malformations. Chi-squared test was used to compare the proportion of children with comorbidities between age groups (0–2 months vs ≥3 months).

In a sensitivity analysis, the outcome definition was expanded to include ICD-10 code B97·4 (Respiratory syncytial virus as the cause of diseases classified elsewhere). Additionally, for the variable “having a sibling hospitalized for LRTI before the age of 4” we restricted the analysis to children with at least one sibling. For “multiple birth”, a sensitivity analysis was conducted among full-term multiple births only.

Data analysis was performed in Stata version 18·0 (StataCorp, College Station, TX, USA).

### Ethics approval

The study was approved by the Swedish Ethical Review Authority (DNR 2018/1697–31/1, amendments DNR 2020–02638 and DNR 2022-05010-0). Participants were automatically included in the registers managed by the National Board of Health and Welfare and Statistics Sweden, and informed consent was not required for this study.

### Role of the funding source

The funders had no role in the study design, data collection, data analysis, data interpretation, writing of the manuscript, or the decision to submit it for publication.

## Results

**Fig. 1:**
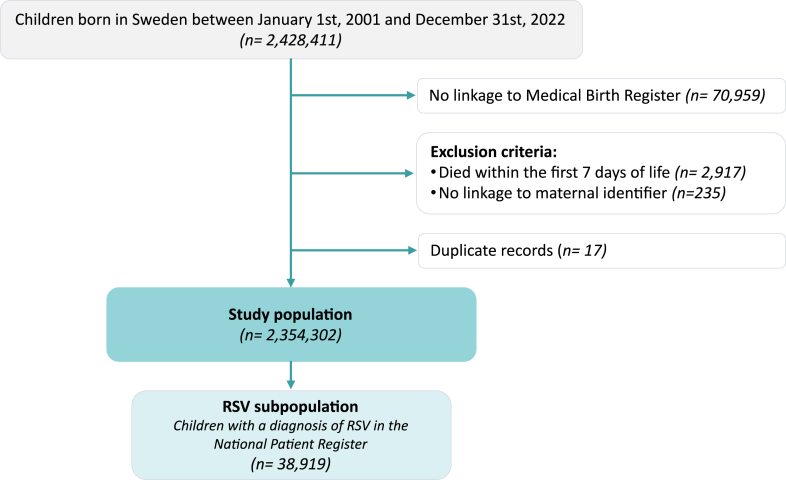
**Flowchart of inclusion in the study.** RSV, Respiratory Syncytial Virus.

A total of 2,428,411 children were born in Sweden during the study period. Children were excluded if they lacked linkage to the MBR (*n* = 70,959, 2·92%), if they died within the first 7 days of life (*n* = 2917, 0·12%), or if they lacked linkage to the maternal identifier (*n* = 235, 0·01%). Following duplicate removal (*n* = 17, <0·01%), the final study population consisted of 2,354,302 children. Of these, a total of 38,919 children (1·65%) had an RSV infection diagnosis in the NPR and were included in the subgroup analysis. This corresponds to an incidence rate of 1406 per 100,000 person-years in children under 1 year of age and 36 per 100,000 person-years in children aged ≥1 year. Supplementary Table S4 presents the annual incidence rates before and after 1 year of age, while Supplementary Figure S3 illustrates the yearly number of RSV-related diagnoses among children aged 0–18 years in Sweden from 2001 to 2023, revealing a generally consistent biennial pattern in RSV incidence, with notable disruption during the COVID-19 pandemic period. A flowchart of inclusion is available in Fig. 1. Sociodemographic characteristics of the study population and of the subgroup of children with a record of RSV diagnosis are reported in Table 1.

**Table 1:** Characteristics of the study population and of the subgroup population with a diagnosis of RSV (Respiratory Syncytial Virus) infection in the National Patient Register.

Sociodemographic characteristics	Children with RSV infection	Full cohort
	M (SD)/N (%)	M (SD)/N (%)
Total sample	38,919 (1·7)	2,354,302 (100·0)
Follow-up for death and prolonged hospitalization [years]	12·7 (6·1)	12·7 (6·2)
Follow-up for ICU admission [years]	8·7 (4·8)	8·7 (5·0)
Age at severe RSV associated outcome [months]^a^	2·3 (1·1–7·3)	2·3 (1·1–7·3)
Male gender	21,699 (55·8)	1,210,574 (51·4)
Season of birth		
Spring	6193 (15·9)	623,355 (26·5)
Summer	7291 (18·7)	625,698 (26·6)
Autumn	11,763 (30·2)	558,546 (23·7)
Winter	13,672 (35·1)	546,703 (23·2)
Weight at birth [grams]	3395·7 (718·6)	3514·9 (578·7)
Small for gestational age	1160 (3·2)	51,248 (2·2)
Classification of prematurity according to WHO		
Full term	34,127 (87·7)	2,220,503 (94·3)
Moderate to late preterm	3531 (9·1)	113,796 (4·8)
Very preterm	763 (2·0)	13,266 (0·6)
Extremely preterm	476 (1·2)	6056 (0·3)
Age of the mother		
<25 years	4779 (12·3)	300,976 (12·8)
25–29 years	11,309 (29·1)	711,303 (30·2)
30–34 years	14,044 (36·1)	830,749 (35·3)
≥35 years	8787 (22·6)	511,273 (21·7)
Maternal BMI before pregnancy		
Underweight	820 (2·3)	52,611 (2·4)
Normal weight	20,251 (56·8)	1,274,591 (58·6)
Overweight	9259 (26·0)	557,411 (25·6)
Obese	5342 (15·0)	289,674 (13·3)
Multiple birth	2137 (5·5)	65,606 (2·8)
Maternal smoking during pregnancy	3024 (8·1)	138,364 (6·1)
Any family member with a diagnosis of asthma	8613 (22·1)	385,825 (16·4)
Parental country of birth		
Both parents born in Sweden	27,622 (71·0)	1,610,403 (68·4)
One parent born abroad	5073 (13·0)	328,104 (13·9)
Both parents born abroad	6224 (16·0)	415,795 (17·7)
Highest level of education in any of the parents		
Primary school	2338 (6·4)	121,715 (5·5)
Secondary school	14,154 (38·7)	805,018 (36·5)
University	20,111 (54·9)	1,277,152 (58·0)
Presence of siblings aged <4 years in the household	21,961 (59·6)	876,058 (39·4)
Presence of siblings aged 4–6 years in the household	6559 (17·8)	340,261 (15·3)
Sibling hospitalized for viral LRTI before the age of 4	573 (1·5)	15,259 (0·6)
**Comorbidities**	**N (%)**	**N (%)**

Atrial septal defect or ventricular septal defect diagnosis	927 (2·4)	27,864 (1·2)
Severe congenital heart defect diagnosis	535 (1·4)	9255 (0·4)
Cerebral palsy diagnosis	435 (1·1)	6310 (0·3)
Down syndrome diagnosis	255 (0·7)	2610 (0·1)
Any chronic lung disease diagnosis	957 (2·5)	14,286 (0·6)
Esophageal malformations diagnosis	90 (0·2)	629 (0·0)
Diagnosis of neonatal respiratory problems of the term child	1719 (4·4)	35,540 (1·5)
Any life limiting condition diagnosis	3533 (9·1)	71,271 (3·0)

Data are reported either as numbers (N) with percentages or as means (M) with standard deviations (SD).

ICU, Intensive Care Unit; WHO, World Health organization; BMI, Body Mass Index; LRTI, lower respiratory tract infection.

a Age is presented as median (interquartile range) instead of mean (SD) due to its skewed distribution.

### ICU admission and death

**Fig. 2:**
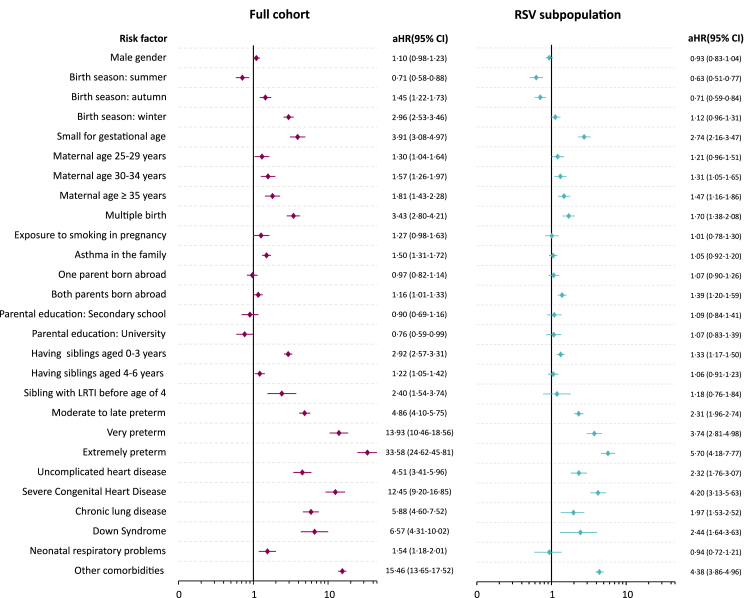
**Hazard ratios (HR) and 95% confidence intervals (CIs) for RSV-related ICU admission or death in the full cohort and among the subpopulation of children with a record of RSV infection.** Other comorbidities: Life-limiting conditions, cerebral palsy, esophageal malformations. Reference categories for categorical variables: Birth season: spring is the reference category; Maternal age: maternal age <25 years is the reference category; Parental birth country: both parents born in Sweden is the reference category; Parental education: primary school is the reference category; Prematurity: full term birth is the reference category. Identified confounders: Sex, parental birth country: none; Older siblings: parental education, parental birth country, maternal age; Multiple birth: parental education, parental birth country, maternal age; Maternal age: parental education, parental birth country; Parental education: parental birth country, maternal age; Small for gestational age: parental education, parental birth country, maternal age, smoking during pregnancy; Maternal smoking during pregnancy: parental education, parental birth country, maternal age; Family history of asthma: parental education, parental birth country; Sibling hospitalized for LRTI before age of 4: parental education, parental birth country, family history of asthma; Congenital Heart Disease: down syndrome, sex, parental education, parental birth country, smoking during pregnancy; Prematurity: down syndrome, sex, congenital heart disease, older siblings (0–3 and 4–7 years), parental education, parental birth country, maternal age, smoking during pregnancy; Chronic lung disease: prematurity, sex, smoking during pregnancy, down syndrome, parental education, congenital heart disease, parental birth country, maternal age; Neonatal respiratory problems of the term child: congenital heart disease, chronic lung disease, small for gestational age, sex, prematurity, multiple birth, smoking during pregnancy; Down Syndrome: parental birth country, maternal age, smoking during pregnancy; Other severe conditions (LLC, cerebral palsy, esophageal malformations): parental birth country, smoking during pregnancy. LRTI, Lower respiratory tract infection; RSV, respiratory Syncytial Virus; ICU, intensive Care Unit.

In the full cohort of children, 1232 experienced RSV-associated ICU admission (*n* = 1210) or death (*n* = 27). Multivariable Cox regression analysis, adjusted for potential confounders (detailed in Supplementary Table S3), identified several factors associated with an increased risk of ICU admission or death. Fig. 2 presents the adjusted hazard ratios (aHR) and 95% CI for all evaluated risk factors for ICU admission or death, both in the full cohort and in the subgroup of children with a record of RSV infection diagnosis (all unadjusted and adjusted HRs are reported in Supplementary Tables S5a and b). The strongest associations were seen for birth in winter (HR 2·96, 95% CI: 2·53–3·46), SGA (aHR 3·91, 95% CI: 3·08–4·97), multiple birth (aHR 3·43, 95% CI: 2·80–4·21), having siblings 0–3 years (aHR 2·92, 95% CI: 2·57–3·31), having a sibling hospitalized for lower respiratory tract infection before age 4 (aHR 2·40, 95% CI: 1·54–3·74) and severe underlying comorbidities. Maternal age, family history of asthma, and having older siblings aged 4–6 years showed a slight increase in risk, while factors such as sex, exposure to smoking during pregnancy, and parental birth country were not significantly associated with the outcome. For variables with non-proportional hazards, a flexible parametric survival model was applied. This analysis showed that the elevated risk persisted throughout childhood for children born prematurely (see Supplementary Figures S4a and b), and for the first two years of life among those born in winter (see Supplementary Figures S4c and d).

In the subgroup analysis including only the children with a recorded RSV infection diagnosis, most factors maintained their association with ICU admission or death, although the HRs were slightly attenuated, particularly for sociodemographic factors. Being born SGA (aHR 2·74, 95% CI: 2·16–3·47), prematurity (very preterm: aHR 3·74, 95% CI: 2·81–4·98; extremely preterm: aHR 5·70, 95% CI: 4·18–7·77) and severe underlying comorbidities retained the strongest HR.

When incorporating the ICD-10 code B97·4 for RSV infection diagnosis in a sensitivity analysis, the results remained consistent in both the full cohort and the RSV subpopulation (see Supplementary Tables S6a and b). The sensitivity analyses conducted among full-term multiple births and among children with at least one sibling yielded similar, though slightly attenuated, associations for both “multiple birth” and “having a sibling hospitalized for LRTI before the age of 4” (see Supplementary Tables S7 and S8).

Table 2 summarizes the characteristics of children who were either admitted to the ICU or died following an RSV infection diagnosis. The median age of those admitted to the ICU was 1·9 months (IQR: 0·9–9·6), and the median length of stay was 46·0 h (IQR: 24·2–97·2). Among these children, 437 (36·1%) received supplemental oxygen via high-flow nasal cannula (HFNC) for a median duration of 18·9 h (IQR: 9·0–34·5); 305 (25·2%) received non-invasive ventilation for a median of 25·3 h (IQR: 12·8–48·5); and 389 (32·1%) received mechanical ventilation for a median of 65·8 h (IQR: 22·8–144·0). The median age of the children who died was 6·6 months (IQR: 2·9–34·3).

**Table 2:** Characteristics and clinical data of the children who had a severe outcome of respiratory syncytial virus infection.

Characteristics	Median (IQR)/N (%)
**a) Death**	
Total sample	27 (100·0)
Age at death [months]	6·6 (2·9–34·3)
Male gender	15 (55·6)
Small for gestational age (Swedish definition)	6 (26·1)
ICU admission	5 (18·5)
Respiratory support with HFNC	0 (0·0)
Respiratory support with CPAP	<5
Mechanical ventilation	<5
Presence of underlying comorbidities^a^	15 (55·6)
**b) ICU admission**	
Total sample	1210 (100·0)
Age at ICU admission [months]	1·9 (0·9–9·6)
Male gender	650 (53·7)
Small for gestational age	84 (7·7)
Duration of stay in the ICU [hours]	46·0 (24·2–97·2)
Non-invasive ventilation while in the ICU	305 (25·2)
Duration of non-invasive ventilation in the ICU [hours]	25·3 (12·8–48·5)
Treatment with HFNO while in the ICU	437 (36·1)
Duration of HFNO treatment in the ICU [hours]	18·9 (9·0–34·5)
Mechanical ventilation in the ICU	389 (32·1)
Duration of mechanical ventilation in the ICU [hours]	65·8 (22·8–144·0)
Presence of underlying comorbidities^a^	500 (41·3)
**c) Prolonged hospitalization**	
Total sample	3766 (100%)
Age at death [months]	2·3 (1·1–6·3)
Length of hospital stay [days]	9·0 (7·0–11·0)
Male gender	2068 (54·9)
Small for gestational age	184 (5·5)
RSV associated PICU/ICU transfer	373 (9·9)
RSV associated respiratory support with HFNC	565 (15·0)
RSV associated respiratory support with CPAP	605 (16·1)
RSV associated mechanical ventilation	138 (3·7)
Presence of underlying comorbidities^a^	1283 (34·1)

Data are reported either as numbers (N) with percentages or as median with interquartile range (IQR). Due to small sample sizes, values <5 are reported as “<5”, and corresponding percentages are not provided.

ICU, Intensive Care Unit; HFNC, High-Flow Nasal Cannula; CPAP, Continuous Positive Airway Pressure; WHO, World health Organization; LLC, Life-limiting conditions.

a Details on comorbidities distribution are provided in Supplementary Table S9.

### Prolonged hospitalization

**Fig. 3:**
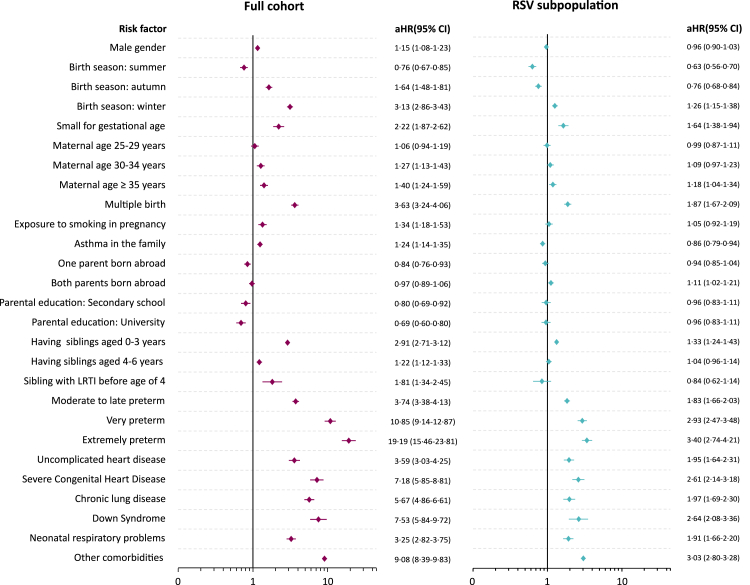
**Hazard ratios (HR) and 95% confidence intervals (CIs) for RSV-related prolonged hospitalization (≥7 days) in the full cohort and among the subpopulation of children with a record of RSV infection.** Other comorbidities: Life-limiting conditions, cerebral palsy, esophageal malformations. Reference categories for categorical variables: Birth season: spring is the reference category; Maternal age: maternal age <25 years is the reference category; Parental birth country: both parents born in Sweden is the reference category; Parental education: primary school is the reference category; Prematurity: full term birth is the reference category. Identified confounders: Sex, parental birth country: none; Older siblings: parental education, parental birth country, maternal age; Multiple birth: parental education, parental birth country, maternal age; Maternal age: parental education, parental birth country; Parental education: parental birth country, maternal age; Small for gestational age: parental education, parental birth country, maternal age, smoking during pregnancy; Maternal smoking during pregnancy: parental education, parental birth country, maternal age; Family history of asthma: parental education, parental birth country; Sibling hospitalized for LRTI before age of 4: parental education, parental birth country, family history of asthma; Congenital Heart Disease: down syndrome, sex, parental education, parental birth country, smoking during pregnancy; Prematurity: down syndrome, sex, congenital heart disease, older siblings (0–3 and 4–7 years), parental education, parental birth country, maternal age, smoking during pregnancy; Chronic lung disease: prematurity, sex, smoking during pregnancy, down syndrome, parental education, congenital heart disease, parental birth country, maternal age; Neonatal respiratory problems of the term child: congenital heart disease, chronic lung disease, small for gestational age, sex, prematurity, multiple birth, smoking during pregnancy; Down Syndrome: parental birth country, maternal age, smoking during pregnancy; Other severe conditions (LLC, cerebral palsy, esophageal malformations): parental birth country, smoking during pregnancy. LRTI, Lower respiratory tract infection; RSV, respiratory Syncytial Virus; ICU, intensive Care Unit.

In the full cohort, a total of 3766 children were hospitalized for ≥7 days following RSV infection. Fig. 3 presents the aHR and 95% CI for all evaluated risk factors for prolonged hospitalization, both in the full cohort and in the subgroup of children with a record of RSV infection diagnosis (all unadjusted and adjusted HRs are reported in Supplementary Tables S9a and b). The risk factors for prolonged hospitalization closely mirrored those for ICU admission or death, including the following: season of birth (winter: HR 3·13, 95% CI: 2·86–3·43), being born SGA (aHR 2·22, 95% CI: 1·87–2·62), multiple birth (aHR 3·63, 95% CI: 3·24–4·06), having older siblings aged 0–3 years (aHR 2·91, 95% CI: 2·71–3·12), and prematurity (very preterm: aHR 10·85, 95% CI: 9·14–12·87; extremely preterm: aHR 19·19, 95% CI: 15·46–23·81). Comorbidities were also associated with an increased risk of prolonged hospitalization, particularly severe CHD (aHR 7·18, 95% CI: 5·85–8·81), Down syndrome (aHR 7·53, 95% CI: 5·84–9·72), and other severe comorbidities (aHR 9·08, 95% CI: 8·39–9·83).

In the subgroup analysis considering only the subpopulation of children with a record of RSV infection, most factors maintained their association with ICU admission or death, although the HRs were slightly attenuated. Family history of asthma (aHR 0·86, 95% CI: 0·79–0·94) was associated with a reduction in the risk of prolonged hospitalization in this subgroup.

When incorporating the diagnostic code B97·4 for RSV infection diagnosis in a sensitivity analysis, the results remained consistent in both the full cohort and the RSV subpopulation (see Supplementary Tables S10a and b). Similar, but slightly attenuated, results were also observed for both “multiple birth” and “having a sibling hospitalized for LRTI before the age of 4” in the sensitivity analysis (see Supplementary Tables S6 and S7).

Table 2 presents the characteristics of children with a prolonged RSV-related hospitalization. The median age of these children was 2·3 months (IQR: 1·1–6·3) and the median hospital stay was 9 days (IQR: 7·0–11·0). Of these, 565 (15·0%) had a record of respiratory support with HFNC, 605 (16·1%) with CPAP, and 138 (3·7%) with mechanical ventilation. Additionally, 373 (9·9%) were transferred to the ICU/PICU.

### Proportion of comorbidities

Underlying comorbidities were seen in 633 (52·3%) of children admitted to the ICU, 1606 (42·6%) of children with a prolonged hospitalization and 21 (77·8%) of children who died (details are available in Supplementary Table S11).

**Fig. 4:**
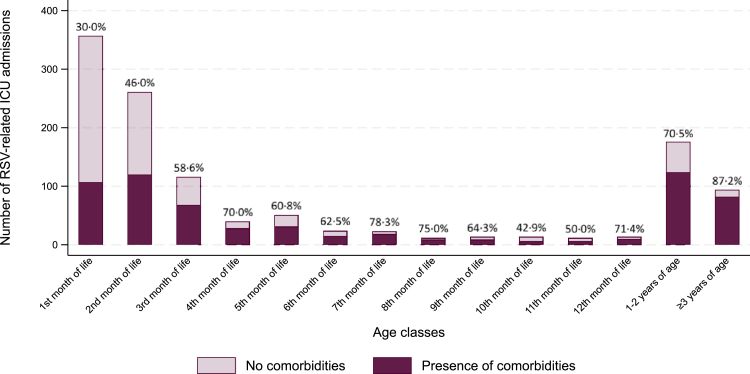
**Prevalence of comorbidities among children with RSV-related ICU admission, classified by age.** Comorbidities include prematurity, Congenital Heart Disease (CHD, both severe CHD and uncomplicated atrial/ventricular septal defects), chronic lung disease, neonatal respiratory conditions of the term child, Trisomy 21, life-limiting conditions (LLC), cerebral palsy, esophageal malformations. Only the first prolonged hospitalization was considered for each child. RSV, Respiratory Syncytial Virus; ICU, Intensive Care Unit.

Fig. 4 shows the proportion of children with comorbidities among those admitted to the ICU or who died, categorized by age. Among these children, the proportion of comorbidities was lower in the first months of life (40·3%, *n* = 298 in those aged 0–2 months) and increased with age. In children aged 3 months and older, 71·6% (*n* = 351) had at least one of the studied comorbidities (*p* < 0·0001 for age-related differences).

**Fig. 5:**
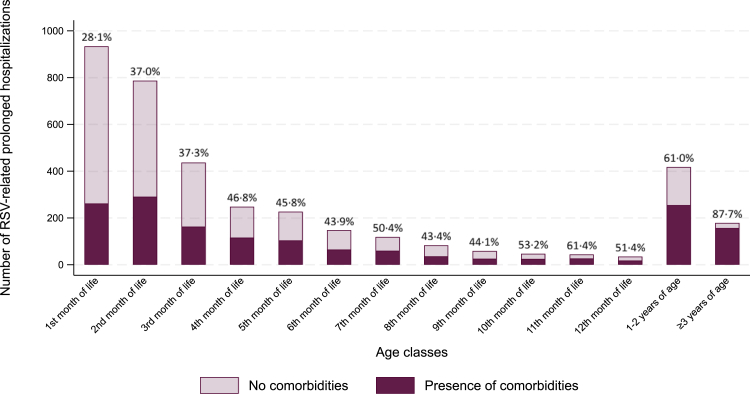
**Prevalence of comorbidities among children with RSV-related prolonged hospitalization (≥7 days), classified by age.** Comorbidities include prematurity, Congenital Heart Disease (CHD, both severe CHD and uncomplicated atrial/ventricular septal defects), chronic lung disease, neonatal respiratory conditions of the term child, Trisomy 21, life-limiting conditions (LLC), cerebral palsy, esophageal malformations. Only the first prolonged hospitalization was considered for each child. RSV, Respiratory Syncytial Virus.

Fig. 5 shows the proportion of children with comorbidities among those with a prolonged hospitalization, categorized by age. Similar to children admitted to the ICU, comorbidities were less common in those aged 0–2 months (33·2%, *n* = 716) and increased with age. Among children older than 3 months, 55·3% (*n* = 889) had at least one underlying condition (*p* < 0·0001 for age-related differences).

## Discussion

In this study, we identified sociodemographic risk factors and comorbidities that are specifically associated with RSV-related death, ICU admission or prolonged hospitalization in children. We report that prematurity, being born SGA, multiple birth, and having underlying comorbidities are the factors most strongly associated with severe RSV disease outcomes.^23^ However, our findings suggest that the majority of children that experience severe adverse outcomes due to RSV infection are previously healthy infants younger than 3 months. This is likely explained, at least in part, by the good coverage of short-acting monoclonal antibodies in high-risk groups, which helps reduce severe outcomes in children with known risk factors.

When analyzing ICU admissions and deaths due to RSV infection, we found that certain sociodemographic factors, such as having siblings aged 0–3 years, having a sibling previously hospitalized due to an LRTI before the age of 4, family history of asthma and being born in autumn or winter (therefore immediately before or during RSV season), were associated with an increased risk of severe disease outcome. When considering siblings with LRTI hospitalization before the age of 4, we performed a sensitivity analysis including only children with siblings of any age to account for the potential confounding effect of simply having a sibling, which confirmed our findings. Previous studies have investigated the role of siblings in the risk of severe RSV, but our study specifically highlights that having siblings aged 0–3 years increases the risk, rather than siblings of any age.^24^ Additionally, our study identifies multiple birth as a strong risk factor for RSV-associated ICU admission, an association that has not been thoroughly explored yet: multiple birth has previously been identified as a possible risk factor for RSV hospitalization, and existing studies also indicate that twins hospitalized with RSV do not have an increased risk of severe disease compared to singletons.^5^^,^^25^ The increased risk may be influenced by prematurity or being born SGA, both of which are more common among multiple births. However, when analyzing the subgroup of full-term multiple births to rule out the possibility that prematurity, rather than multiple birth itself, was the actual risk factor, the risk remained elevated, though slightly weaker. Although the majority of children admitted to ICU were otherwise healthy, 41·3% had at least one underlying comorbidity. This represents a substantial over-representation relative to the prevalence of such conditions in the general pediatric population. These findings suggest that children with comorbidities are at a markedly increased risk of severe RSV requiring intensive care compared to healthy children.

In an attempt to distinguish between risk factors that increase the likelihood of medically attended RSV infection at the population level and those that predict a more severe disease course among infected children, we performed a sub-analysis restricted to children with an RSV diagnosis. As expected, the point estimates for most sociodemographic risk factors were attenuated or non-significant in this analysis. By contrast, prematurity, being born SGA, and comorbidities (except for respiratory conditions in term-born children) remained the primary determinants of severe outcomes, consistent with the existing literature.^9^^,^^26^, ^27^, ^28^ This change suggests that while sociodemographic factors or familial predisposition (such as asthma or LRTI in siblings) primarily contribute to increased risk of exposure or susceptibility to RSV, once infected, a child's intrinsic characteristics play a greater role in determining disease severity.^29^

Risk factors for prolonged hospitalization due to RSV infection appear to follow a similar pattern to those associated with ICU admission and mortality, with certain medical conditions contributing to longer hospital stays. However, as with ICU admission and death, when focusing only on children with a record of RSV infection diagnosis, sociodemographic risk factors became less significant, while prematurity, SGA, and underlying comorbidities remained key determinants of long hospital stays. Particularly, children with heart diseases were found to have a higher risk of prolonged hospitalization, consistent with the findings by Resch et al., who reported longer hospital and ICU stays, as well as an increased need for respiratory support in this group.^30^

While underlying comorbidities and prematurity are known risk factors for severe RSV infection,^4^^,^^5^^,^^31^ our study also shows that majority of newborns and infants with severe disease outcomes were previously healthy and born at term. Specifically, among infants younger than 3 months, 59·8% of those who died or required ICU admission, and 66·8% of those hospitalized for ≥7 days fell into this group. In contrast, comorbidities play a more significant role in severe outcomes among older children.

The major strength of our study was the use of national health and population registers, which provided extensive, high-quality coverage of the full Swedish population ensuring a high degree of representativeness. The large study population including over 2·3 million children allowed us to investigate rare outcomes that are impossible to assess in smaller studies due to statistical power limitations. Furthermore, the nationwide, longitudinal nature of the data enabled a comprehensive assessment of risk factors for severe RSV disease over a 23-year period, enhancing the generalizability of our findings to other high-income settings with similar health care systems. Notably, to our knowledge, this is the first study to analyze nationwide data on the risk of severe outcomes due to RSV infection, addressing a critical gap in the literature: in fact, while most research focuses on risk factors for hospitalization, our study examined the most severe outcomes, providing valuable insights for pediatric research and public health planning. One limitation of our study is the potential variation in diagnostic practices for RSV over the study period and across different regions, particularly with less consistent testing in older children and those with milder illness, which could have introduced bias. Additionally, we could not account for clustering of patients within hospitals due to incomplete data on hospital affiliation for children without the outcome. Nevertheless, as our focus was on the most severe outcomes, where extensive diagnostic workups have consistently been performed, this was likely not a major source of bias. Another potential limitation of this study is the lack of clinical data, which hampered our possibility to accurately characterize disease severity. Instead, our outcome definition relied on register data and could have been influenced by factors such data availability or hospital policies. While we likely underestimated the overall RSV incidence, we believe that we accurately captured the RSV associated events most crucial to prevent (i.e., prolonged hospitalization, ICU admission and death). We also retrieved data on respiratory support during hospitalization, which provided valuable insight into the clinical condition of the children and partially compensated for the lack of detailed clinical data. Another potential limitation of this study is the lack of ICU data prior to 2008, resulting in an underestimation of the total burden of RSV-related ICU admissions; however, this does not affect the validity of our risk estimates, as time-at-risk for the ICU admission analysis was calculated from 2008 onwards. Furthermore, not all children undergo RSV testing, which may result in the subgroup of children with an RSV diagnosis in the NPR not being fully representative, potentially including younger and more fragile children. Finally, some children may have multiple risk factors, making it difficult to determine the direction of associations: the use of DAGs and multivariable analysis, however, contributed to minimize the risk of confounding and bias.

In conclusion, this study highlights that several factors, including comorbidities, are associated with severe RSV outcomes. However, the majority of severe events occur in previously healthy, full-term infants, particularly those under three months of age. If the goal is to effectively reduce the most severe RSV-related outcomes through immunization, our findings suggest that relying solely on the presence of severe comorbidities to guide eligibility may be insufficient. In fact, we identified additional risk factors, including being born small for gestational age, multiple birth, and having siblings aged 0–3 years, that are not currently included in RSV immunization policies but may confer increased vulnerability to severe outcomes of RSV infection. A comprehensive immunization strategy that broadens eligibility criteria to include these factors, combined with public health intervention targeting parents, may be essential to more effectively protect the most vulnerable infants.

## Contributors

SR conceptualized the study, had a leading role in the funding and supervision of all stages of the study. GD participated in the study design and register-linkages, had a leading role in the data management, performed the statistical analyses and drafted the first version of the manuscript. CL participated in the study design, had a leading role in the register-linkages and data management and supervised the statistical analyses. AS and ECO participated in the study design and had a leading role in the register-linkages. PV, SH, and TA provided valuable input on the study design and data analysis. CA conceptualized the study, had a leading role in the study design, and register linkage and provided funding for the study. GD, CL, AS, ECO, CA, and SR had full access to the data. GD, CL, and SR have accessed and verified the data. All authors critically revised the manuscript, provided comments, and approved the final version for submission.

## Data sharing statement

Original data are held by Swedish National Board of Health and Welfare, Statistics Sweden, and the Swedish Intensive Care Registry. Due to Swedish data storage laws we cannot make the data publicly available. However, any researcher can access the data by obtaining an ethical approval from a regional ethical review board and thereafter asking the registers for the original data. Pseudonymized data may be provided upon requests to the corresponding author, if providing a reasonable proposal and if an appropriate data sharing agreement with Karolinska Institutet can be established.

## Declaration of interests

Giulia Dallagiacoma reports a research grant from the Martin Rind Foundation. Cecilia Lundholm reports a travel grant from the Swedish Heart-Lung Foundation. Pekka Vartiainen reports single consultancy fees from GSK and Pfizer, lecture fee from Sanofi, and Grants from Päivikki and Sakari Sohlberg Foundation and Pediatric Research Foundation in Finland. Santtu Heinonen is employed by FVR-Finnish Vaccine Research, which conducts vaccine research funded by several vaccine manufacturers. He reports having received a one-time consultancy fee from MSD/Merck, unrelated to his current role at FVR. As part of his employment at FVR, he has participated in advisory boards for MSD, but has not received any personal remuneration for this participation. Samuel Rhedin reports research grants from Ake Wiberg foundation, the Society of Child Welfare, Karolinska Institutet, and Region Stockholm, single consultancy fees from Biomerieux and MSD. Catarina Almqvist reports research grants from the Swedish Research Council, the Swedish Heart-Lung Foundation, the Swedish Asthma and Allergy Association Research Fund, Region Stockholm, and the Foundation Frimurare Barnhuset in Stockholm. Awad Smew, Emma Caffrey Osvald, and Tobias Alfvén report no conflicts of interest.

## Appendix A Supplementary data

Swedish Abstract

## References

[1] Li Y., Wang X., Blau D.M. (2022). Global, regional, and national disease burden estimates of acute lower respiratory infections due to respiratory syncytial virus in children younger than 5 years in 2019: a systematic analysis. Lancet.

[2] Del Riccio M., Spreeuwenberg P., Osei-Yeboah R. (2023). Burden of Respiratory Syncytial Virus in the European Union: estimation of RSV-associated hospitalizations in children under 5 years. J Infect Dis.

[3] Hamrin J., Bennet R., Berner J., Rotzen-Ostlund M., Eriksson M. (2021). Rates and risk factors of severe respiratory syncytial virus infection in 2008-2016 compared with 1986-1998. Acta Paediatr.

[4] Vartiainen P., Jukarainen S., Rhedin S.A. (2023). Risk factors for severe respiratory syncytial virus infection during the first year of life: development and validation of a clinical prediction model. Lancet Digit Health.

[5] Haerskjold A., Kristensen K., Kamper-Jorgensen M., Nybo Andersen A.M., Ravn H., Graff Stensballe L. (2016). Risk factors for hospitalization for respiratory syncytial virus infection: a population-based cohort study of Danish children. Pediatr Infect Dis J.

[6] Lim S.A., Chan M., Hu N. (2024). Risk factors and clinical prognosis associated with RSV-ALRI intensive care unit admission in children <2 years of age: a multicenter study. Pediatr Infect Dis J.

[7] Fraser L.K., Miller M., Hain R. (2012). Rising national prevalence of life-limiting conditions in children in England. Pediatrics.

[8] Cai W., Buda S., Schuler E., Hirve S., Zhang W., Haas W. (2020). Risk factors for hospitalized respiratory syncytial virus disease and its severe outcomes. Influenza Other Respir Viruses.

[9] Scheltema N.M., Gentile A., Lucion F. (2017). Global respiratory syncytial virus-associated mortality in young children (RSV GOLD): a retrospective case series. Lancet Glob Health.

[10] Ares-Gomez S., Mallah N., Santiago-Perez M.I. (2024). Effectiveness and impact of universal prophylaxis with nirsevimab in infants against hospitalisation for respiratory syncytial virus in Galicia, Spain: initial results of a population-based longitudinal study. Lancet Infect Dis.

[11] Carbajal R., Boelle P.Y., Pham A. (2024). Real-world effectiveness of nirsevimab immunisation against bronchiolitis in infants: a case-control study in Paris, France. Lancet Child Adolesc Health.

[12] Drysdale S.B., Cathie K., Flamein F. (2023). Nirsevimab for prevention of hospitalizations due to RSV in infants. N Engl J Med.

[13] Phijffer E.W., de Bruin O., Ahmadizar F. (2024). Respiratory syncytial virus vaccination during pregnancy for improving infant outcomes. Cochrane Database Syst Rev.

[14] Simões E.A.F., Madhi S.A., Muller W.J. (2023). Efficacy of nirsevimab against respiratory syncytial virus lower respiratory tract infections in preterm and term infants, and pharmacokinetic extrapolation to infants with congenital heart disease and chronic lung disease: a pooled analysis of randomised controlled trials. Lancet Child Adolesc Health.

[15] NT-rådet (2025). Beyfortus (nirsevimab) för prevention av sjukdom orsakad av respiratoriskt syncytialvirus (RSV).

[16] Folkhälsomyndigheten (2024). Allvarlig RS-virusinfektion bland barn och vuxna i Sverige-Sammanfattande kunskapsunderlag för riskgruppsdefinition och förebyggande insatse.

[17] Warmington A.V., Bowdish D.M.E., Sherifali D., Sloboda D.M. (2024). A scoping review of the relationship between maternal BMI and offspring incidence of respiratory infection: where do we go from here?. AJPM Focus.

[18] Maršál K., Persson P.H., Larsen T., Lilja H., Selbing A., Sultan B. (1996). Intrauterine growth curves based on ultrasonically estimated foetal weights. Acta Paediatr.

[19] Hviid M.M., Skovlund C.W., Morch L.S., Lidegaard O. (2017). Maternal age and child morbidity: a Danish national cohort study. PLoS One.

[20] World Health Organization (2024). Born Too Soon: Decade of Action on Preterm Birth.

[21] Ortqvist A.K., Lundholm C., Wettermark B., Ludvigsson J.F., Ye W., Almqvist C. (2013). Validation of asthma and eczema in population-based Swedish drug and patient registers. Pharmacoepidemiol Drug Saf.

[22] Lambert P. (2023). STPM3: Stata Module to Fit Flexible Parametric Survival Models. S459207 ed.

[23] Deng S., Cong B., Edgoose M., De Wit F., Nair H., Li Y. (2024). Risk factors for respiratory syncytial virus-associated acute lower respiratory infection in children under 5 years: an updated systematic review and meta-analysis. Int J Infect Dis.

[24] Havdal L.B., Boas H., Bekkevold T. (2022). Risk factors associated with severe disease in respiratory syncytial virus infected children under 5 years of age. Front Pediatr.

[25] Dotan M., Ashkenazi-Hoffnung L., Samra Z. (2013). Hospitalization for respiratory syncytial virus bronchiolitis and disease severity in twins. Isr Med Assoc J.

[26] Curns A.T., Rha B., Lively J.Y. (2024). Respiratory syncytial virus-associated hospitalizations among children <5 years old: 2016 to 2020. Pediatrics.

[27] Mitchell I., Defoy I., Grubb E. (2017). Burden of respiratory syncytial virus hospitalizations in Canada. Can Respir J.

[28] Shi T., Vennard S., Mahdy S., Nair H., investigators R. (2022). Risk factors for poor outcome or death in young children with respiratory syncytial virus-associated acute lower respiratory tract infection: a systematic review and meta-analysis. J Infect Dis.

[29] Goetghebuer T., Kwiatkowski D., Thomson A., Hull J. (2004). Familial susceptibility to severe respiratory infection in early life. Pediatr Pulmonol.

[30] Resch B., Kurath-Koller S., Hahn J., Raith W., Kostenberger M., Gamillscheg A. (2016). Respiratory syncytial virus-associated hospitalizations over three consecutive seasons in children with congenital heart disease. Eur J Clin Microbiol Infect Dis.

[31] Shmueli E., Goldberg O., Mei-Zahav M. (2021). Risk factors for respiratory syncytial virus bronchiolitis hospitalizations in children with chronic diseases. Pediatr Pulmonol.

