# Supplementary material for: Risk factors for severe outcomes of respiratory syncytial virus infection in children: a nationwide cohort study in Sweden
Source: Lancet Reg Health Eur. 2025 Sep 9;58:101447. doi: 10.1016/j.lanepe.2025.101447 (PMC12624800; doi:10.1016/j.lanepe.2025.101447)
Supplement: Swedish Abstract [file mmc1.docx]

***This translation in Swedish was submitted by the authors and we reproduce it as supplied. It has not been peer reviewed. Our editorial processes have only been applied to the original abstract in English, which should serve as reference for this manuscript.***

**SAMMANFATTNING**
**Bakgrund:**  Riskfaktorer för sjukhusinläggning till följd av infektion med respiratoriskt syncytialvirus (RSV) hos barn är väl etablerade, men få studier har undersökt allvarliga sjukdomsutfall av RSV. Syftet med denna studie var att undersöka riskfaktorer för allvarlig RSV-infektion hos barn i åldern 0–18 år.

**Metod:** Vi genomförde en registerbaserad kohortstudie som inkluderade alla barn födda i Sverige mellan 2001 och 2022. Data på ICD-10-diagnoser, sociodemografiska faktorer och samsjuklighet inhämtades från nationella hälso- och populationsregister. Huvudutfall var död, intensivvårdsinläggning (IVA) och långvarig sjukhusvistelse (≥7 dagar) till följd av RSV. Justerade riskkvoter (aHR) och 95 % konfidensintervall (KI) beräknades med multivariat Cox-regressionsanalys, både i kohorten i sin helhet samt i en begränsad population av barn som erhållit en RSV-diagnos.

**Resultat:** Av 2 354 302 barn erhöll 38 919 (1,7%) en RSV-diagnos. Av de senare hade 4 621 (11,9%) ett allvarligt sjukdomsförlopp. Medianåldern för barn som lades in på IVA var 1.9 månader, och 500 (41,3%) hade en underliggande grundsjukdom. Följande faktorer var starkast kopplade till IVA-inläggning eller död i hela kohorten: födsel under vintern (HR 2,96; 95% KI: 2,53–3,46), liten för graviditetslängden (aHR 3,91; 95% KI: 3,08–4,97), flerbördsfödsel (aHR 3,43; 95% KI: 2,80–4,21), syskon i åldern 0–3 år (aHR 2,92; 95% KI: 2,57–3,31) samt underliggande grundsjukdom (aHR >4). Liknande, men något svagare, samband sågs när endast barn med RSV-diagnos undersöktes. Underliggande grundsjukdom var mindre vanligt bland barn under tre månader med allvarlig sjukdom jämfört med äldre barn (40,3 % vs 71,6 %, p<0,0001).

**Betydelse:** Allvarlig RSV-infektion drabbar i hög grad friska, fullgångna spädbarn under tre månaders ålder, även om den individuella risken är högst bland dem med svår underliggande grundsjukdom. Riskfaktorer såsom liten för graviditetslängden, flerbördsfödsel och äldre syskon i förskoleålder ingår idag inte i strategier för RSV-immunisering, men bör övervägas för att bättre kunna förebygga allvarlig sjukdom.

**Finansiering:** Finansiellt stöd till studien erhölls från Karolinska Institutet (KID-medel), Vetenskapsrådet, Hjärt-Lungfonden, Astma- och Allergiförbundet, Region Stockholm, Strategiska forskningsområdet i epidemiologi vid Karolinska Institutet, Sällskapet Barnavård, Åke Wibergs stiftelse, Martin Rinds stiftelse och Stiftelsen Frimurare Barnhuset i Stockholm.
