# Supplementary material for: Risk factors for severe outcomes of respiratory syncytial virus infection in children: a nationwide cohort study in Sweden
Source: Lancet Reg Health Eur. 2025 Sep 9;58:101447. doi: 10.1016/j.lanepe.2025.101447 (PMC12624800; doi:10.1016/j.lanepe.2025.101447)
Supplement: Supplementary Materials [file mmc2.pdf]

SUPPLEMENTARY MATERIAL

TABLE OF CONTENTS

Supplementary Material – Swedish National Health and Population Registers used 3

Supplementary Figures 4

Figure S1. Data availability from the included registers across the study period. 4

Figure S2. Directed Acyclic Graphs 5

Figure S2a - Directed Acyclic Graph (DAG) illustrating the relationship between having older siblings and severe RSV infection 5

Figure S2b - Directed Acyclic Graph (DAG) illustrating the relationship between twins and severe RSV infection..5

Figure S2c - Directed Acyclic Graph (DAG) illustrating the relationship between maternal age and severe RSV infection..6

Figure S2d - Directed Acyclic Graph (DAG) illustrating the relationship between parental education and severe RSV infection 6

Figure S2e - Directed Acyclic Graph (DAG) illustrating the relationship between small for gestational age and severe RSV infection 7

Figure S2f - Directed Acyclic Graph (DAG) illustrating the relationship between exposure to smoking during pregnancy and severe RSV infection. 7

Figure S2g - Directed Acyclic Graph (DAG) illustrating the relationship between family history of asthma and severe RSV infection 8

Figure S2h - Directed Acyclic Graph (DAG) illustrating the relationship between having a sibling who was hospitalized for lower respiratory tract infection before the age of 4 and severe RSV infection..8

Figure S2j - Directed Acyclic Graph (DAG) illustrating the relationship between prematurity and severe RSV infection..9

Figure S2m - Directed Acyclic Graph (DAG) illustrating the relationship between trisomy 21 and severe RSV infection..11

Figure S2n - Directed Acyclic Graph (DAG) illustrating the relationship between other severe comorbidities and severe RSV infection 11

Figure S3. Cases of Respiratory Syncytial Virus (RSV) diagnosis among children in Sweden during the study period (2021-2023)...12

Figure S4. Flexible Parametric Survival Model Analysis 13

Figure S4a. Flexible parametric Model for the risk of RSV-related death or ICU admission in preterm children over time, according to premature class from the 8th day of life until 18 years of age. 13

Figure S4b. Flexible parametric Model for the risk of death or ICU admission in preterm children over time, according to premature class from the 8th day of life until 5 years of age. 13

Figure S4c. Flexible Parametric Model for the risk of RSV-related death or ICU admission in children according to birth season, from the 7th day of life until 5 years of age. The presented HR unadjusted, as no potential confounders were identified....................................14

Figure S4d. Flexible Parametric Model for the risk of RSV-related death or ICU admission in children according to birth season, from the 7th day of life until 2 years of age. The presented HR unadjusted, as no potential confounders were identified....................................14

Supplementary Material - Algorithm for asthma diagnosis 15

Supplementary Tables 16

Table S1. ICD-10 codes used for comorbidities 16

Table S2. ICD-10 (version 2016) diagnostic coding framework used to identify and categorise children with life-limiting conditions (Fraser et al.). 16

Table S3. Potential confounders identified through Directed Acyclic Graphs. 17

Table S4. Annual incidence of Respiratory Syncytial Virus (RSV) diagnosis in Sweden among children 0-18 years. 18

Table S5a. Unadjusted and adjusted Hazard Ratios (HR) and Confidence Intervals (CI) for RSV-associated ICU admission or death in the full cohort..19

Table S5b. Unadjusted and adjusted Hzard Ratios (HR) and Confidence Intervals (CI) for RSV-associated ICU admission or death in the RSV subpopulation 20

Table S6a. Sensitivity Analysis - Unadjusted and adjusted Hazard Ratios (HR) and Confidence Intervals (CI) for RSV-associated ICU admission or death in the full cohort including ICD-10 code B97.4...21

Table S6b. Sensitivity Analysis - Unadjusted and adjusted Hazard Ratios (HR) and Confidence Intervals (CI) for RSV-associated ICU admission or death in the RSV subpopulation including ICD-10 code B97.4 22

Table S7. Sensitivity Analysis - Unadjusted and adjusted Hazard Ratios (HR) and Confidence Intervals (CI) for the variable "Multiple birth" only conducted among full-term twins...........23

Table S8. Sensitivity Analysis - Unadjusted and adjusted Hazard Ratios (HR) and Confidence Intervals (CI) for the variable "having a sibling hospitalized for LRTI before the age of 4" only conducted among children with a sibling...23

Table S9a. Unadjusted and adjusted Hazard Ratios (HR) and Confidence Intervals (CI) for RSV-associated prolonged hospitalization in the full cohort...24

Table S9b. Unadjusted and adjusted Hazard Ratios (HR) and Confidence Intervals (CI) for RSV-associated prolonged hospitalization in the RSV subpopulation 25

Table S10a. Sensitivity Analysis - Unadjusted and adjusted Hazard Ratios (HR) and Confidence Intervals (CI) for RSV-associated prolonged hospitalization in the full cohort including ICD-10 code B97.4...26

Table S10b. Sensitivity Analysis - Unadjusted and adjusted Hazard Ratios (HR) and Confidence Intervals (CI) for RSV-associated prolonged hospitalization in the RSV subpopulation including ICD-10 code B97.4 27

Table S11. Prevalence of underlying comorbidities by outcome group 28

Supplementary Material – Swedish National Health and Population Registers used

1. **Total Population Register (TPR)**
   Maintained by Statistics Sweden, the TPR includes data on life events such as birth, death, marital status, family relationships, and migration. It is updated daily via the Swedish Tax Agency and allows for longitudinal tracking of the population.
   Reference: Ludvigsson JF, Almqvist C, Bonamy AK, et al. Registers of the Swedish total population and their use in medical research. *Eur J Epidemiol*. 2016;**31**(2):125–136

2. **National Patient Register (NPR)**
   Maintained by the National Board of Health and Welfare, the NPR includes data on all inpatient care since 1987 and most specialized outpatient visits since 2001. It contains information on diagnoses, procedures, and hospital visits.
   Reference: Ludvigsson JF, Andersson E, Ekbom A, et al. External review and validation of the Swedish national inpatient register. *BMC Public Health*. 2011 Jun 9;**11**:450.

3. **Medical Birth Register (MBR)**
   Maintained by the National Board of Health and Welfare, the MBR includes data on pregnancies, deliveries, and neonatal outcomes since 1973 and covers about 98% of all births in Sweden. It includes information from antenatal, delivery, and neonatal care, recorded by midwives and physicians.
   Reference : Cnattingius S, Källén K, Sandström A, et al. The Swedish medical birth register during five decades: documentation of the content and quality of the register. *Eur J Epidemiol*. 2023;**38**(1):109–120.

4. **Swedish Prescribed Drug Register (SPDR)**
   Maintained by the National Board of Health and Welfare, the SPDR contains data on all prescribed and dispensed medications in Sweden since July 1, 2005. It includes information such as the Anatomical Therapeutic Chemical (ATC) classification code, defined daily doses, prescriber information, and date of dispensing.
   Reference: Wettermark B, Hammar N, Fored CM, et al. The new Swedish Prescribed Drug Register-opportunities for pharmacoepidemiological research and experience from the first six months. *Pharmacoepidemiol Drug Saf*. 2007;**16**(7):726–735

5. **Cause of Death Register**
   Maintained by the National Board of Health and Welfare, this register provides data on date and cause of death (underlying and contributing causes) for all deceased Swedish residents since 1952. Overall, 96% of individuals in the cause of death register have a specific underlying cause of death recorded.
   Reference: Brooke HL, Talbäck M, Hörnblad J, et al. The Swedish cause of death register. *Eur J Epidemiol*. 2017 Sep;**32**(9):765–773

6. **Longitudinal Integration Database for Health Insurance and Labour Market Studies (LISA)**
   Maintained by Statistics Sweden, it includes sociodemographic data such as education, income, employment, and family structure. It covers the adult Swedish population aged ≥ 16 years registered on December 31 each year since 1990. Participation in Swedish government-administered registers such as LISA is compulsory, and hence selection bias is minimized. Education data are available in > 98% of all individuals aged 25-64 years.
   Reference: Ludvigsson JF, Svedberg P, Olén O, Bruze G, Neovius M. The longitudinal integrated database for health insurance and labour market studies (LISA) and its use in medical research. *Eur J Epidemiol*. 2019;**34**(4):423–437

7. **Multi-Generation Register (MGR)**
   Maintained by Statistics Sweden, the MGR contains provides data on both biological and adoptive parents, allowing to identify full- and half- siblings as well. Maternal information is available for 97% and paternal information for 95% of index individuals born from 1932 onwards (that were alive on January 1, 1961).
   Reference: Ekbom A. The Swedish Multi-generation Register. *Methods Mol Biol*. 2011;**675**:215–220

8. **Swedish Intensive Care Registry (SIR)**
   This is a national quality register that includes data on admissions to intensive care units (ICUs) across Sweden. It contains detailed clinical information such as diagnoses, interventions, medical parameters, length of stay, and outcomes. The registry covers both adult and pediatric ICUs (the latter from 2008 onward).
   Reference: The Swedish Intensive Care Registry (SIR). https://www.icuregswe.org (accessed on June 25, 2025)

Supplementary Figures

Figure S1. Data availability from the included registers across the study period.

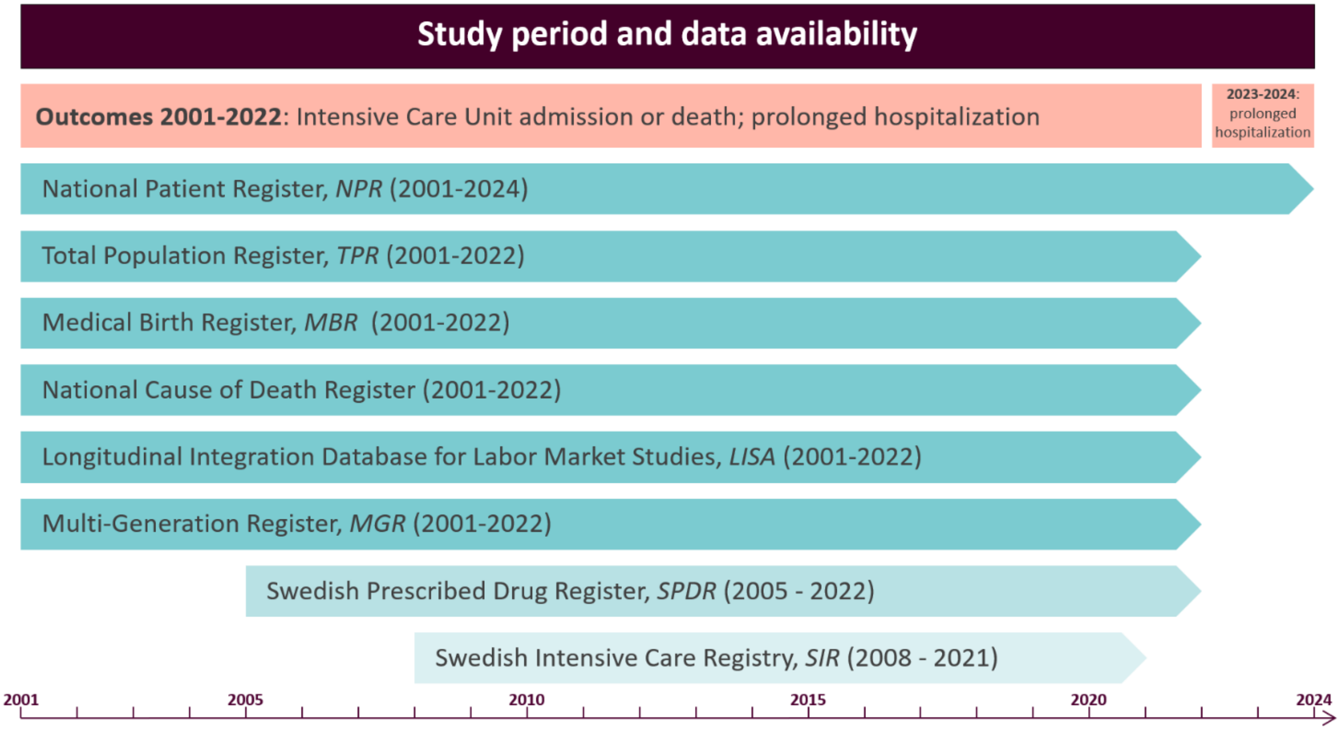

Figure S1. *Data availability from the included registers across the study period*

Figure S2. Directed Acyclic Graphs

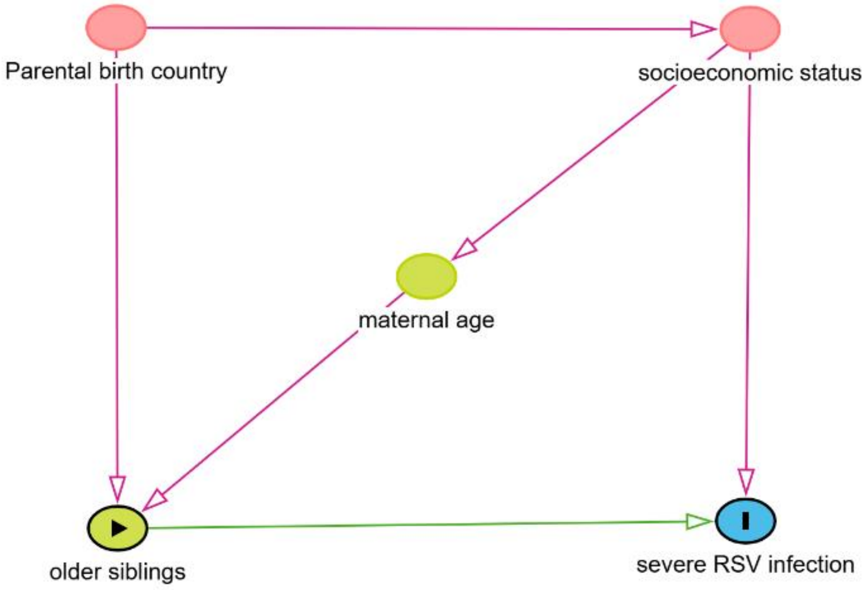

Figure S2a - Directed Acyclic Graph (DAG) illustrating the relationship between having older siblings and severe RSV infection

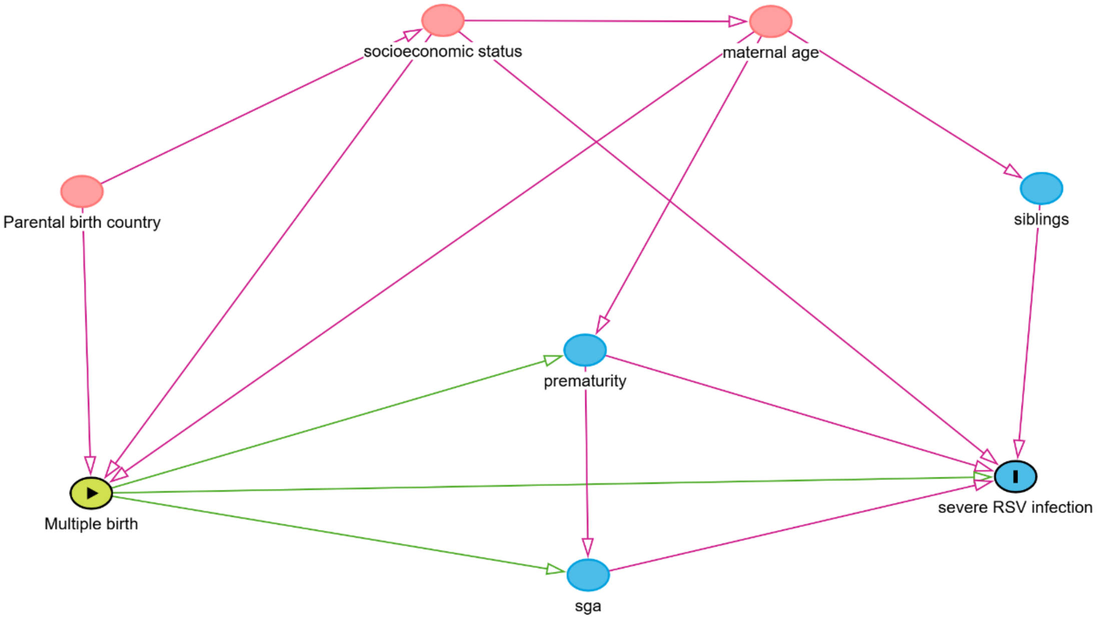

Figure S2b - Directed Acyclic Graph (DAG) illustrating the relationship between twins and severe RSV infection

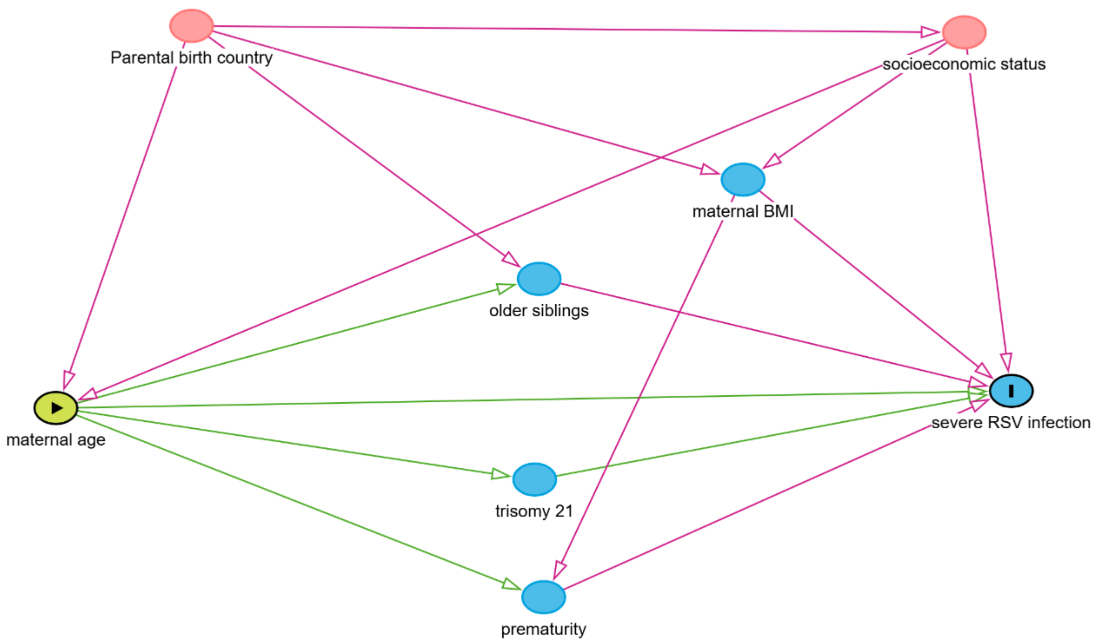

Figure S2c - Directed Acyclic Graph (DAG) illustrating the relationship between maternal age and severe RSV infection

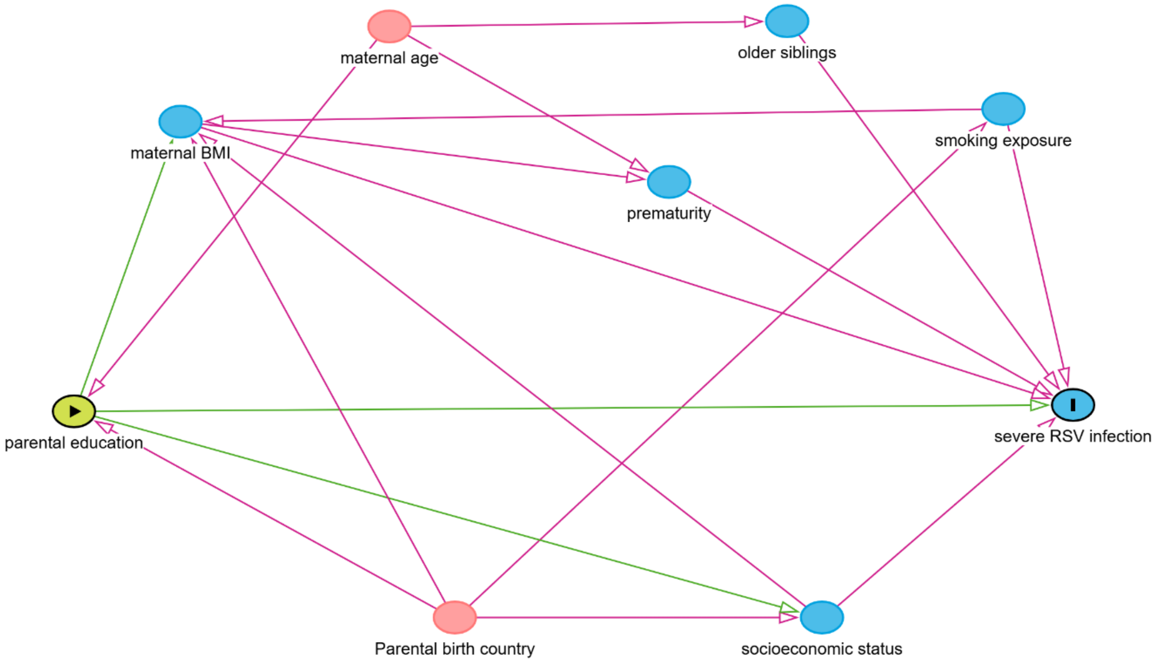

Figure S2d - Directed Acyclic Graph (DAG) illustrating the relationship between parental education and severe RSV infection

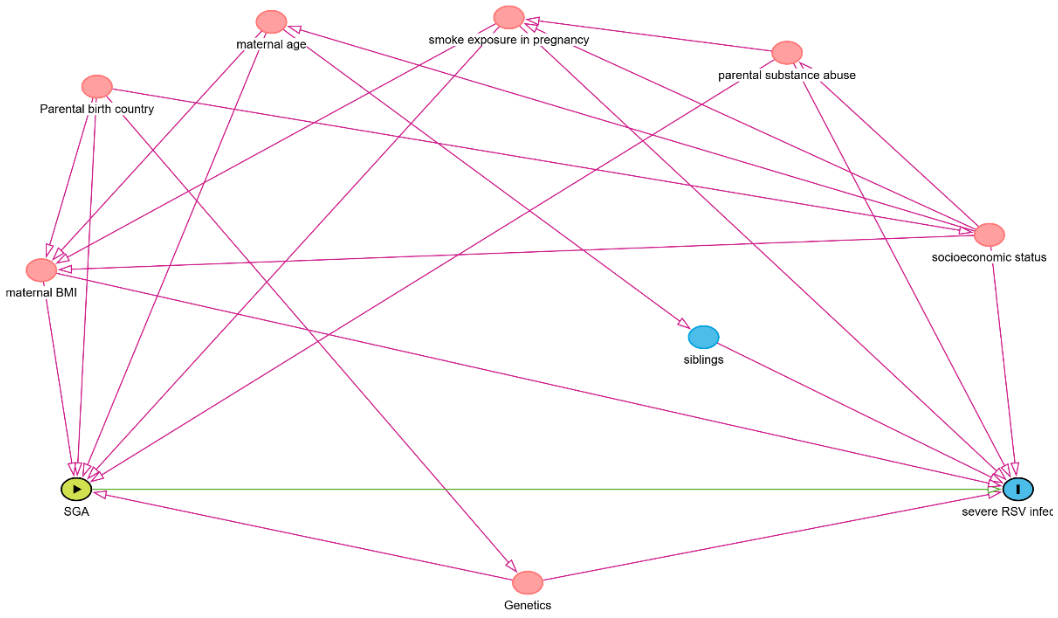

Figure S2e - Directed Acyclic Graph (DAG) illustrating the relationship between small for gestational age and severe RSV infection

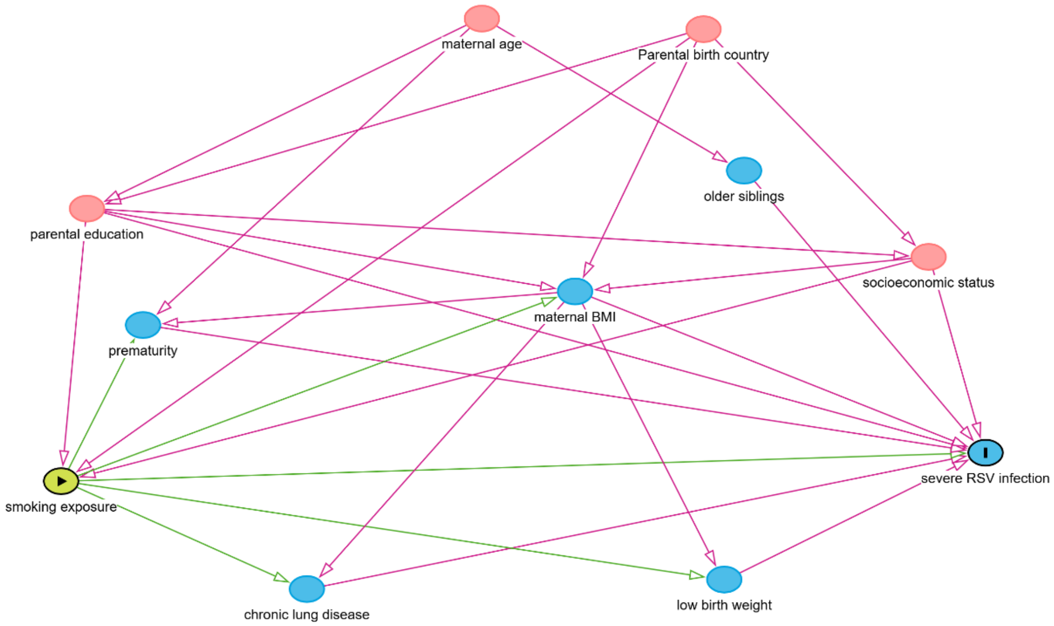

Figure S2f - Directed Acyclic Graph (DAG) illustrating the relationship between exposure to smoking during pregnancy and severe RSV infection.

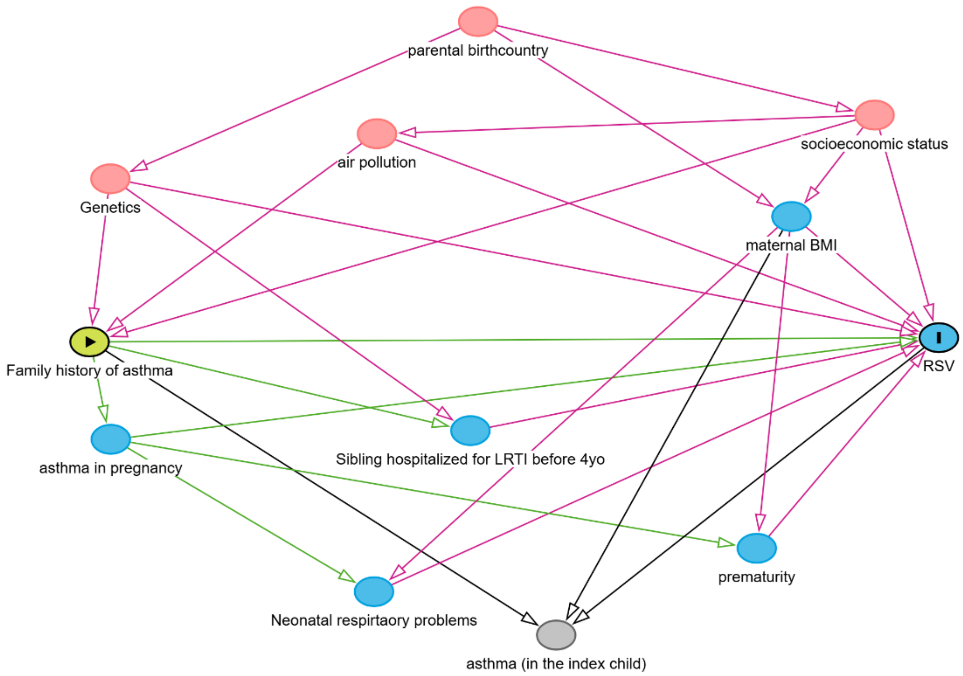

Figure S2g - Directed Acyclic Graph (DAG) illustrating the relationship between family history of asthma and severe RSV infection

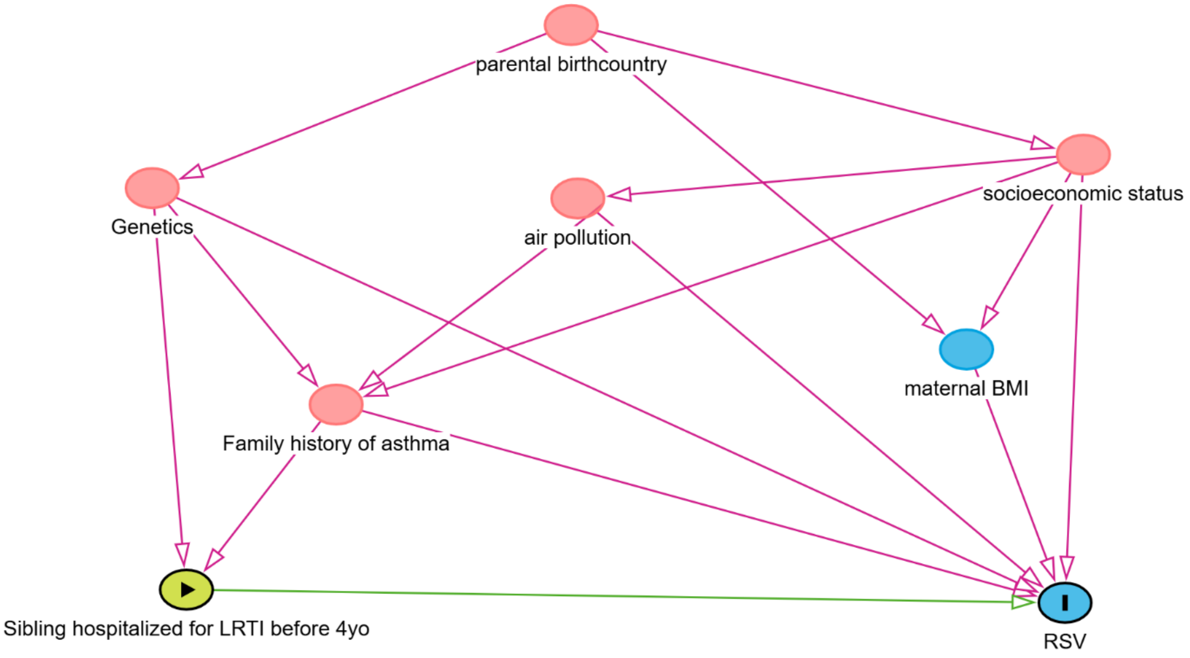

Figure S2h - Directed Acyclic Graph (DAG) illustrating the relationship between having a sibling who was hospitalized for lower respiratory tract infection before the age of 4 and severe RSV infection

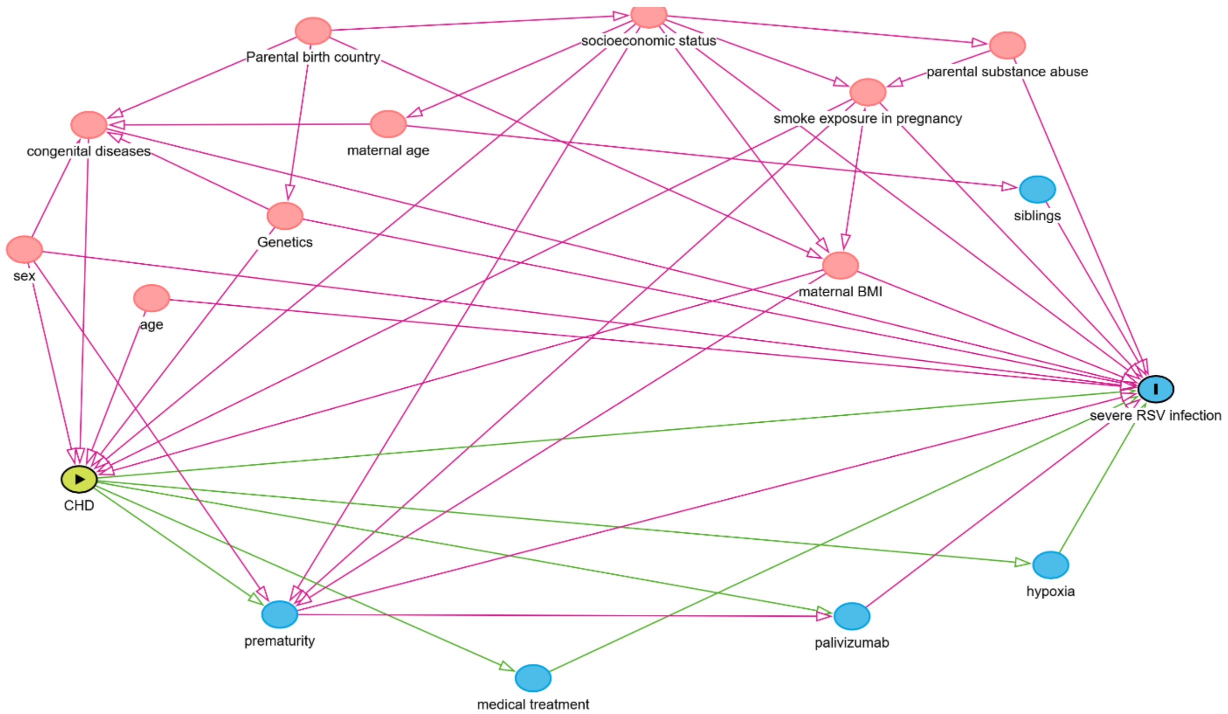

Figure S2i - Directed Acyclic Graph (DAG) illustrating the relationship between congenital heart disease and severe RSV infection

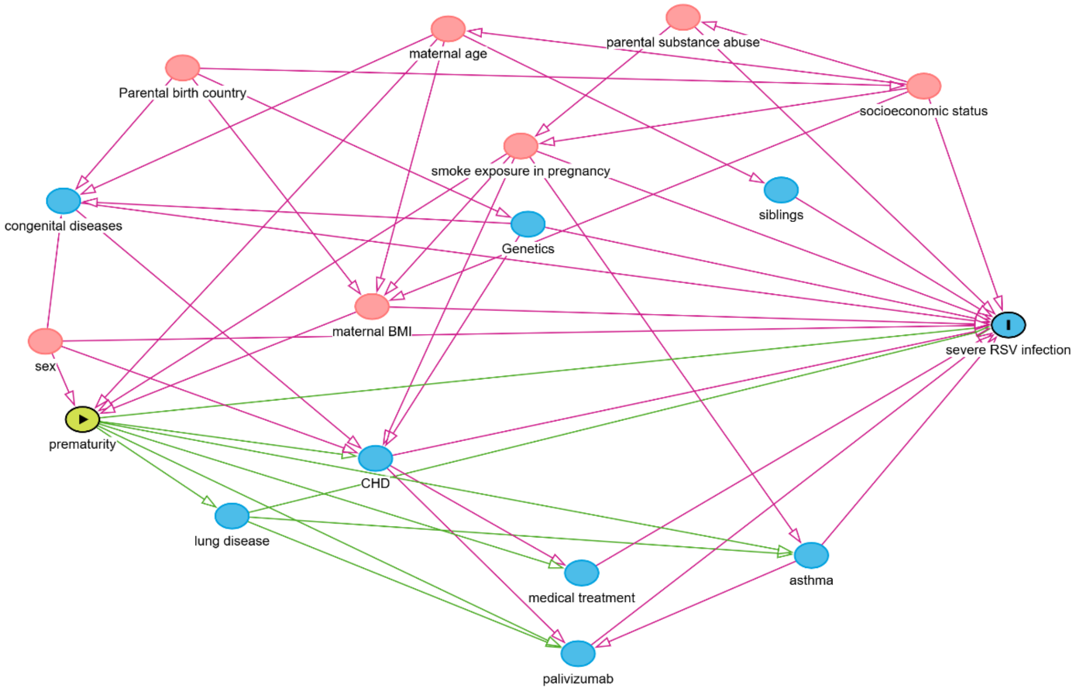

Figure S2j - Directed Acyclic Graph (DAG) illustrating the relationship between prematurity and severe RSV infection

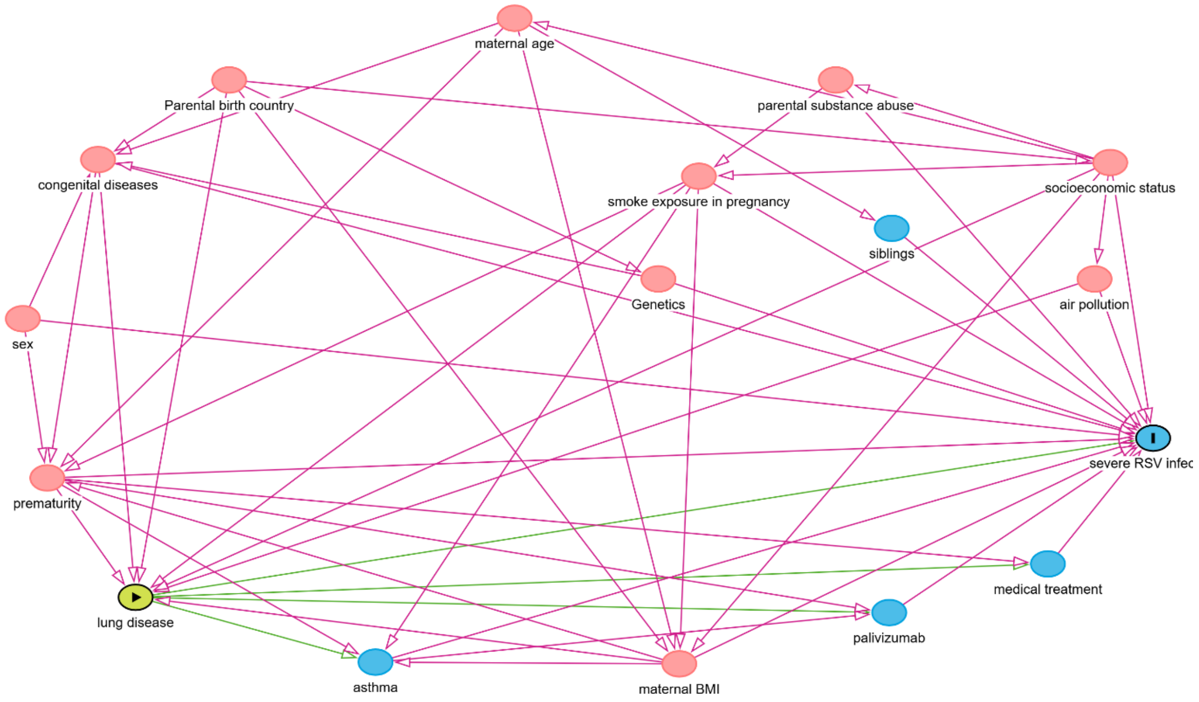

Figure S2k - Directed Acyclic Graph (DAG) illustrating the relationship between chronic lung disease and severe RSV infection

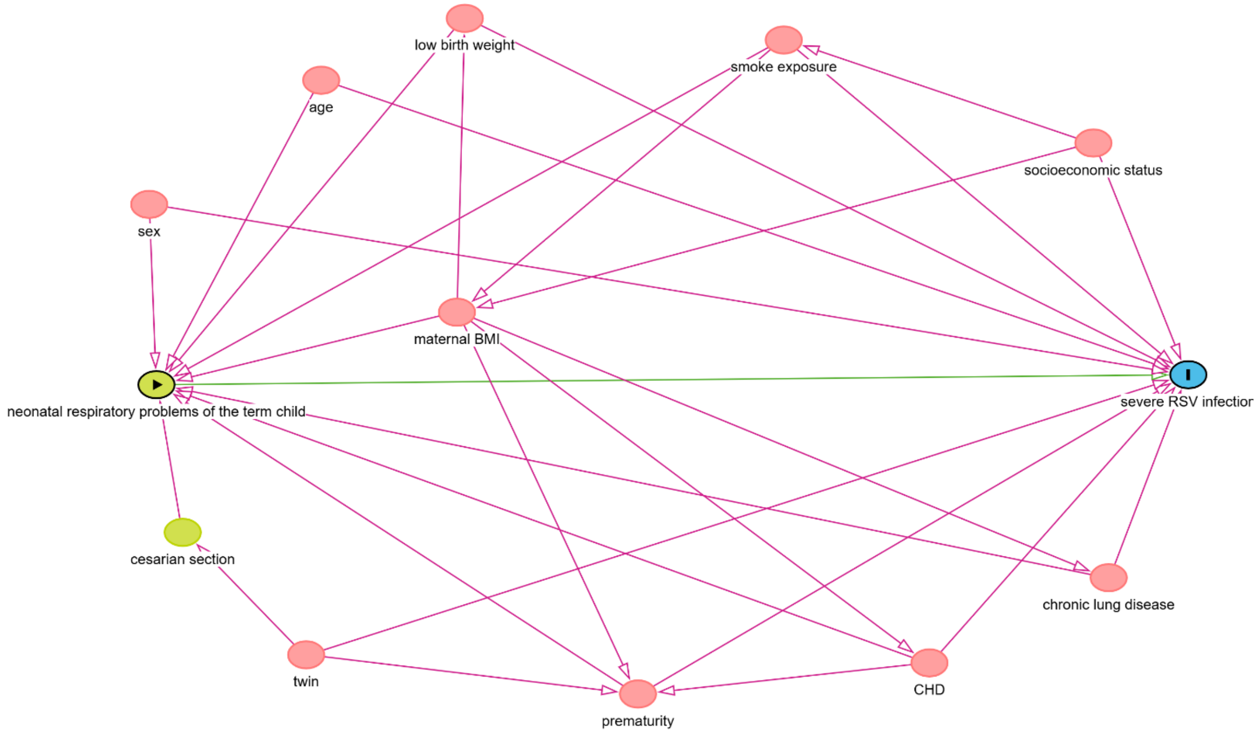

Figure S2l - Directed Acyclic Graph (DAG) illustrating the relationship between neonatal respiratory problems of the term child and severe RSV infection

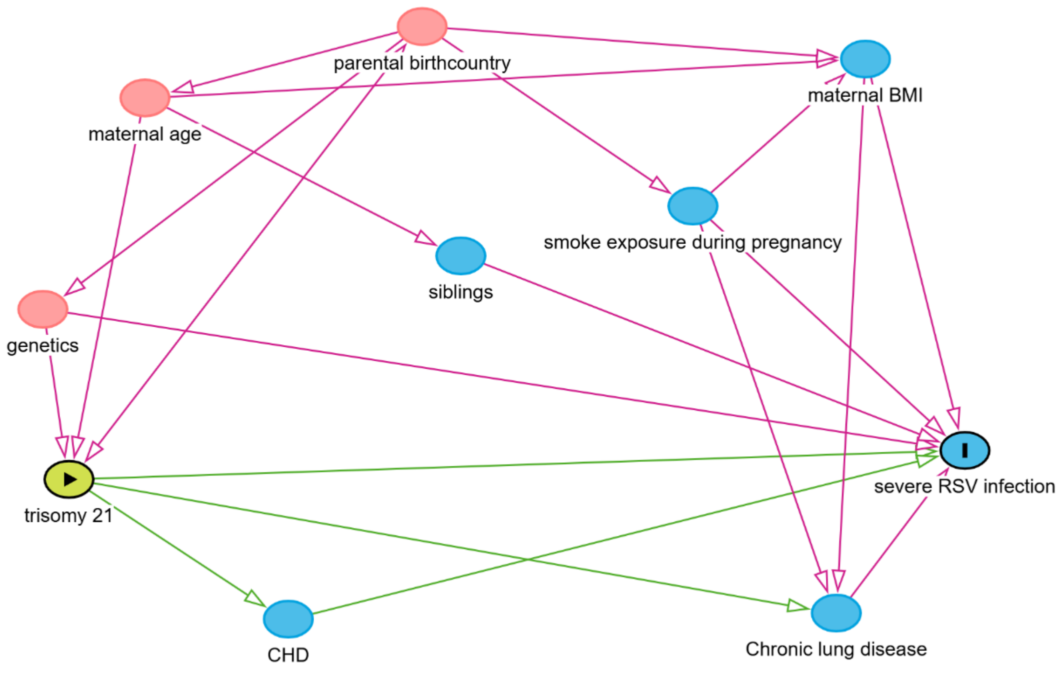

Figure S2m - Directed Acyclic Graph (DAG) illustrating the relationship between trisomy 21 and severe RSV infection

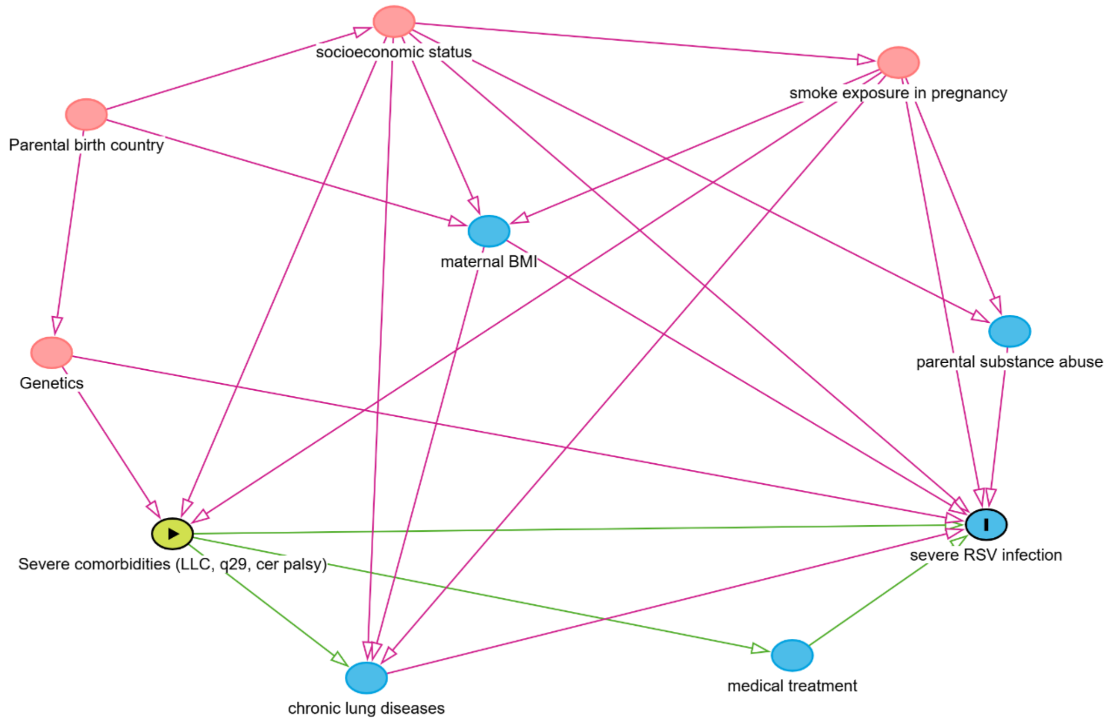

Figure S2n - Directed Acyclic Graph (DAG) illustrating the relationship between other severe comorbidities and severe RSV infection

Figure S3. Cases of Respiratory Syncytial Virus (RSV) diagnosis among children in Sweden during the study period (2021-2023)

RSV, respiratory Syncytial Virus; LRTI, Lower respiratory tract Infection.

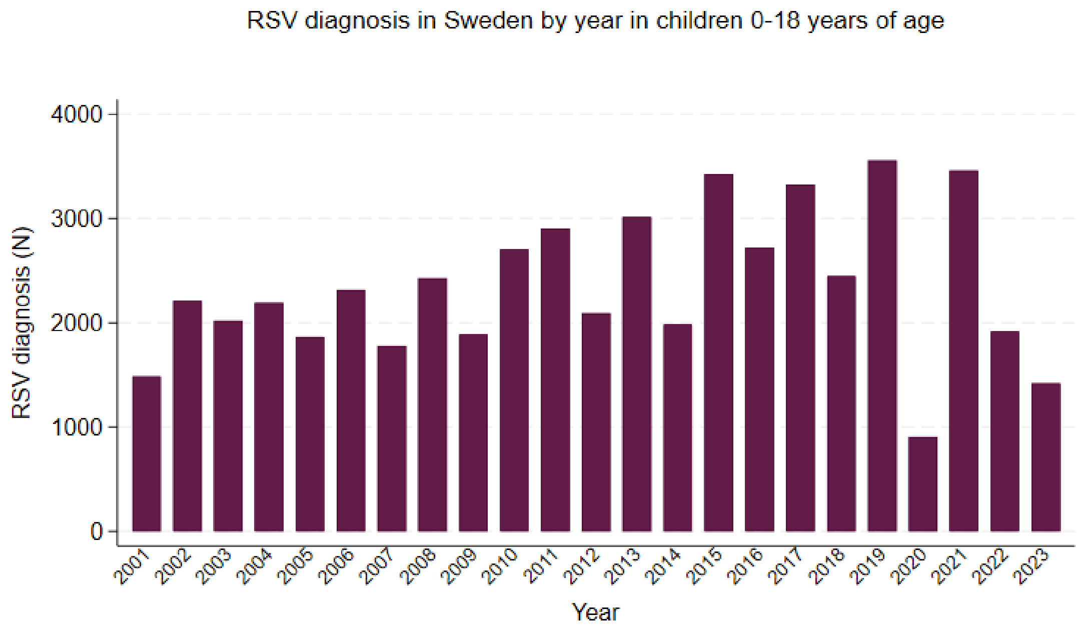

Figure S4. Flexible Parametric Survival Model Analysis

Flexible parametric survival models were fitted with varying numbers and placements of knots to assess sensitivity to spline specification. We ultimately selected 3 degrees of freedom for both the baseline hazard and time-varying effects. Alternative models tested produced similar curves or had convergence problems, supporting the robustness of our results.

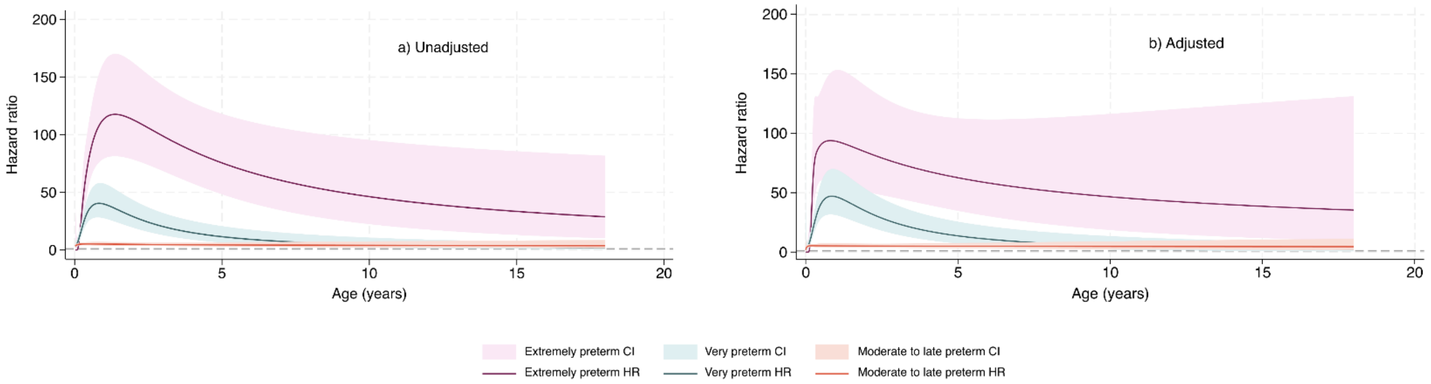

Figure S4a. Flexible parametric Model for the risk of RSV-related death or ICU admission in preterm children over time, according to premature class from the 8th day of life until 18 years of age.

HR, Hazard ratio; CI, Confidence Interval; RSV, Respiratory Syncytial Virus; ICU, Intensive Care Unit.

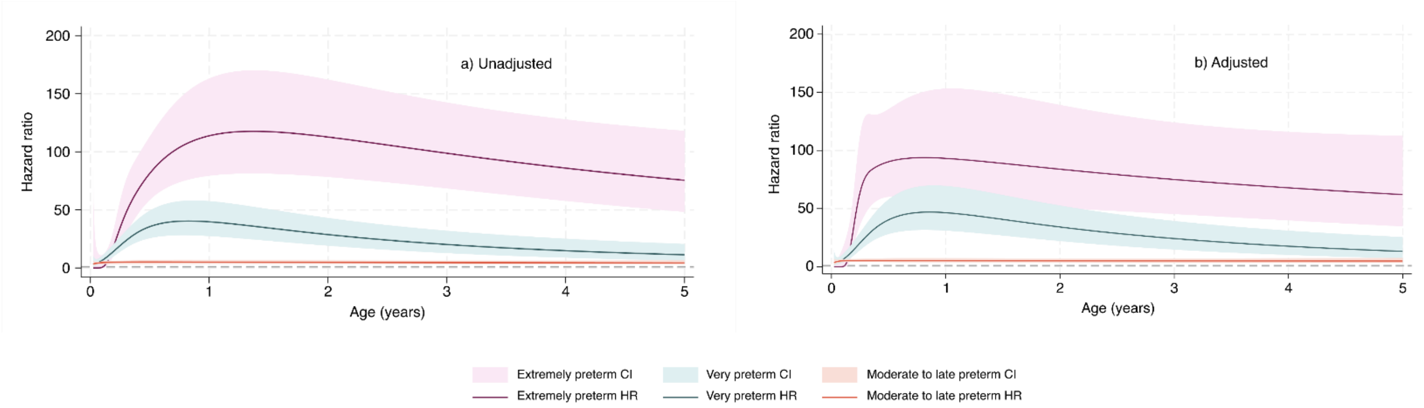

Figure S4b. Flexible parametric Model for the risk of death or ICU admission in preterm children over time, according to premature class from the 8th day of life until 5 years of age.

HR, Hazard ratio; CI, Confidence Interval; RSV, Respiratory Syncytial Virus; ICU, Intensive Care Unit.

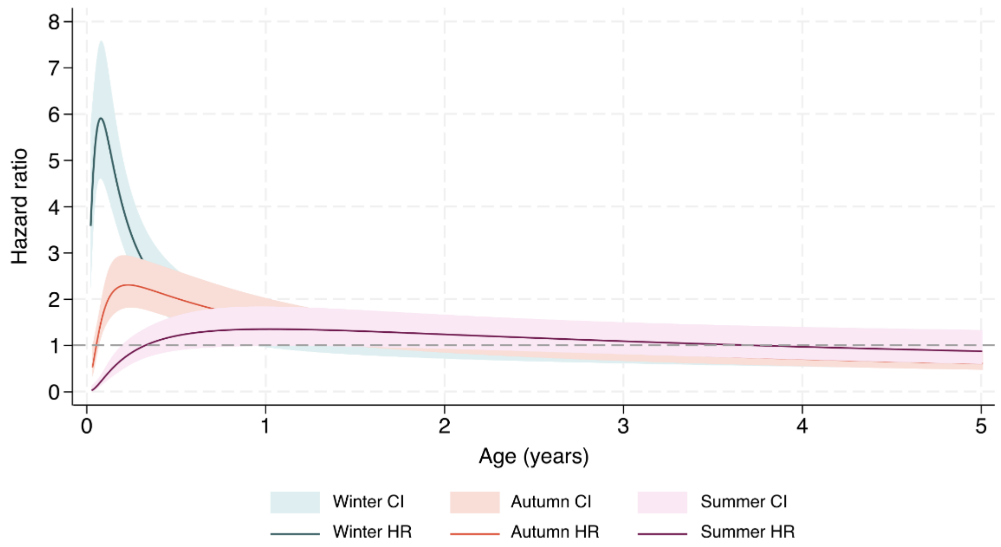

Figure S4c. Flexible Parametric Model for the risk of RSV-related death or ICU admission in children according to birth season, from the 7th day of life until 5 years of age. The presented HR unadjusted, as no potential confounders were identified

HR, Hazard ratio; CI, Confidence Interval; RSV, Respiratory Syncytial Virus; ICU, Intensive Care Unit.

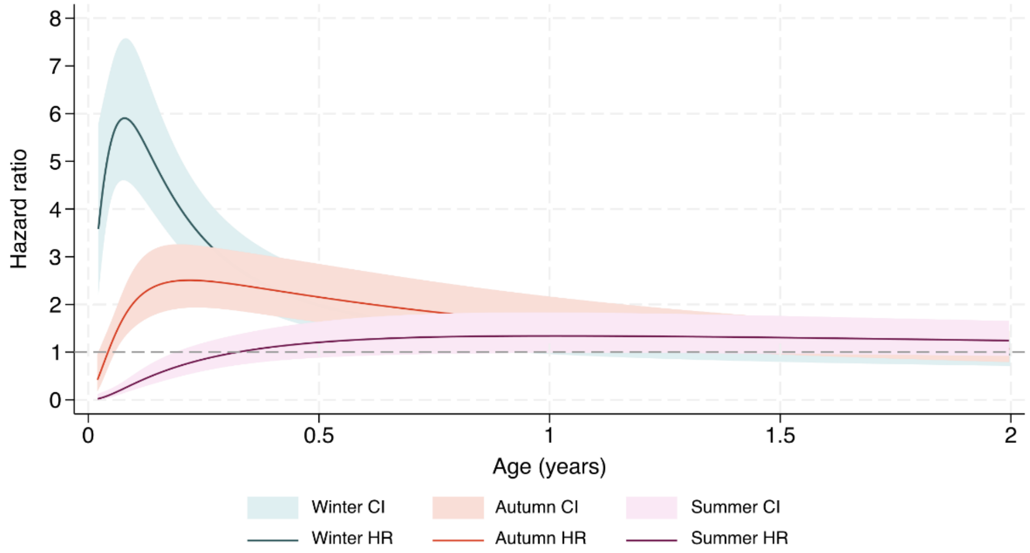

Figure S4d. Flexible Parametric Model for the risk of RSV-related death or ICU admission in children according to birth season, from the 7th day of life until 2 years of age. The presented HR unadjusted, as no potential confounders were identified.

HR, Hazard ratio; CI, Confidence Interval; RSV, Respiratory Syncytial Virus; ICU, Intensive Care Unit.

Supplementary Material - Algorithm for asthma diagnosis

The presence of asthma in parents or older siblings of index children was identified using a previously validated algorithm developed by Örtqvist et al. (20), based on national registry data.

A) Before the age of 4.5 years:

- Fulfilling either of the two medication criteria based on the Swedish Prescribed Drug Register (SPDR, see detailed criteria below)

AND

- Having a diagnosis of asthma in the National Patient Register (NPR):
  - ICD-10: J45–J46
  - ICD-9: 493
  - ICD-8: 493
  - ICD-7: 241

B) After the age of 4.5 years:

- Fulfilling either of the two medication criteria based on the SPDR (see detailed criteria below)

AND/OR

- Having a diagnosis of asthma in the NPR (same ICD codes as above)

C) If not fulfilling the criteria in either of the two age intervals:
Asthma may still be classified as present if:

- Fulfilling either of the two medication criteria based on the PDR across age periods,

AND

- Having a diagnosis of asthma in the NPR before age 4.5 years

Medication Criteria for Asthma Ever (from the SPDR):
1) General criterion:
≥2 dispensations of any of the following:
- Inhaled corticosteroids (ICS): R03BA
- Leukotriene receptor antagonists (LTRA): R03DC03
- Combination β2-agonist + ICS (B2_ICS): R03AK

→ For **children below 4.5 years of age**, at least 14 days must separate the two dispensations.

2) Alternative criterion:
≥3 dispensations within 12 months of any of the following:
- ICS (R03BA)
- LTRA (R03DC03)
- B2_ICS (R03AK)
- Inhaled β2-agonists (short- and long-acting):
  R03AC02, R03AC03, R03AC12, R03AC13

Supplementary Tables

Table S1. ICD-10 codes used for comorbidities

Atrial Septal Defect: Q21.1 AND no Q2 (other than Q25.0, Patent ductus arteriosus)

Ventricular Septal Defect: Q21.0 AND no Q2 (other than Q25.0, Patent ductus arteriosus)

Severe Congenital Heart Disease: Q200, Q201, Q202, Q203, Q204, Q205, Q208, Q209, Q212, Q213, Q214, Q218, Q220, Q222, Q223, Q224, Q226, Q228, Q232, Q234, Q238, Q243, Q244, Q245, Q251, Q252, Q254, Q255, Q257, Q258, Q262)

Down syndrome : Q90

Oesophageal atresia : Q39.0 or Q39.1

Neonatal respiratory distress: P22, P24

Chronic Lung Diseases :

-**Bronchopulmonary displasia**: P27.1

-Interstitial lung disorders : J70.4, J84.1, J84.9

-Congenital lung malformations : Q31-Q33

Cerebral palsy : G80-G83 (except for those included in LLC: G800 G808 G823 G824 G825)

Table S2. ICD-10 (version 2016) diagnostic coding framework used to identify and categorise children with life-limiting conditions (Fraser et al.).

Neurology: A17 A810 A811 F803 F842 G10 G111 G113 G12 G20 G230 G238 G318 G319 G35 G404 G405 G600 G601 G702 G709 G710 G711 G712 G713 G800 G808 G823 G824 G825 G934 G936 G937

Haematology: B20 B21 B22 B23 B24 D561 D610 D619 D70 D761 D81 D821 D83 D891

Oncology: C D444 D48 (Central Nervous System: C70,C71,C72, D33, D43)

Metabolic: E310 E348 E702 E71 E72 E74 E75 E76 E77 E791 E830 E880 E881

Respiratory : E84 J841 J96 J984

Circulatory: I21 I270 I42 I613 I81

Gastrointestinal: K550 K559 K72 K74 K765 K868

Genitourinary: N17 N18 N19 N258 (Early stage (1-3)

Renal: N181, N182, N183)

Perinatal: P101 P112 P210 P285 P290 P293 P350 P351 P358 P371 P524 P525 P529 P832 P912 P916 P960

Congenital: Q000 Q01 Q031 Q039 Q040 Q042 Q043 Q044 Q046 Q049 Q070 Q200 Q203 Q204 Q206 Q208 Q213 Q232 Q218 Q220 Q221 Q224 Q225 Q226 Q230 Q234 Q239 Q254 Q256 Q262 Q264 Q268 Q282 Q321 Q336 Q396 Q410 Q419 Q437 Q442 Q445 Q447 Q601 Q606 Q614 Q619 Q642 Q743 Q748 Q750 Q772 Q773 Q774 Q780 Q785 Q792 Q793 Q804 Q81 Q821 Q824 Q858 Q860 Q870 Q871 Q872 Q878 Q91 Q920 Q921 Q924 Q927 Q928 Q932 Q933 Q934 Q935 Q938 Q952

Other : H111 H498 H355 M313 M321 M895 T860 T862 Z515

Table S3. Potential confounders identified through Directed Acyclic Graphs.

***Other severe conditions: Life-limiting conditions, oesophageal malformations, cerebral palsy.**

Corresponding figure S2	Risk factor	Confounders
	**Sex, parental birth country**	none
Figure S2.a	**Older siblings**	Parental education, parental birth country, maternal age
Figure S2.b	**Twins**	Parental education, parental birth country, maternal age
Figure S2.c	**Maternal age**	Parental education, parental birth country
Figure S2.d	**Parental education**	Parental birth country, maternal age
Figure S2.e	**Small for gestational age**	Parental education, parental birth country, maternal age, smoking during pregnancy, maternal BMI
Figure S2.f	**Exposure to smoking during pregnancy**	Parental education, parental birth country, maternal age
Figure S2.g	**Family history of asthma**	Parental education, parental birth country
Figure S2.h	**Sibling hospitalized for LRTI before age of 4**	Parental education, parental birth country, family history of asthma
Figure S2.i	**CHD**	Down syndrome, sex, parental education, parental birth country, smoking during pregnancy, maternal BMI
Figure S2.j	**Prematurity**	Down syndrome, sex, congenital heart disease, older siblings (0-3 and 4-6 years), parental education, parental birth country, maternal age, smoking during pregnancy, maternal BMI
Figure S2.k	**Lung disease**	Prematurity, sex, smoking during pregnancy, Down syndrome, parental education, congenital heart disease, parental birth country, maternal age, maternal BMI
Figure S2.l	**Neonatal respiratory problems of the term child**	congenital heart disease, chronic lung disease, small for gestational age, sex, prematurity, multiple birth, smoking during pregnancy, maternal BMI
Figure S2.m	**Trisomy 21**	Parental birth country, maternal age, smoking during pregnancy
Figure S2.n	**Other severe conditions***	Parental birth country, smoking during pregnancy

Table S4. Annual incidence of Respiratory Syncytial Virus (RSV) diagnosis in Sweden among children 0-18 years.

RSV, Respiratory Syncytial Virus.

* Population of Sweden reported by age by Statistikmyndigheten (Statistics Sweden, SCB)
** Incidence rate per 100,000 person-years

Year	Total RSV cases (0-18 years)	RSV cases <1 year	Population* <1 year	Incidence** <1 year	RSV cases ≥ 1 year	Population* ≥1 year	Incidence** ≥1 year
**2001**	1,487	1,062	91,653	1,159	425	1,946,626	22
**2002**	2,212	1,741	96,026	1,813	471	1,947,637	24
**2003**	2,020	1,556	99,230	1,568	464	1,949,762	24
**2004**	2,193	1,853	101,090	1,833	340	1,949,108	17
**2005**	1,864	1,624	101,555	1,599	240	1,946,092	12
**2006**	2,315	2,049	106,278	1,928	266	1,948,713	14
**2007**	1,779	1,574	107,757	1,461	205	1,948,945	11
**2008**	2,429	2,127	109,664	1,940	302	1,948,223	16
**2009**	1,887	1,645	112,120	1,467	242	1,941,740	12
**2010**	2,705	2,361	115,910	2,037	344	1,934,019	18
**2011**	2,906	2,504	112,114	2,233	402	1,932,934	21
**2012**	2,091	1,759	113,487	1,550	332	1,936,323	17
**2013**	3,014	2,601	114,008	2,281	413	1,952,816	21
**2014**	1,986	1,627	115,880	1,404	359	1,977,540	18
**2015**	3,426	2,876	115,878	2,482	550	2,014,114	27
**2016**	2,721	2,300	119,023	1,932	421	2,064,315	20
**2017**	3,328	2,713	116,614	2,326	615	2,114,144	29
**2018**	2,449	1,988	116,839	1,701	461	2,149,597	21
**2019**	3,558	2,715	115,383	2,353	843	2,175,451	39
**2020**	906	694	113,589	611	212	2,189,794	10
**2021**	3,459	2,025	114,663	1,766	1,434	2,200,558	65
**2022**	1,915	955	105,186	908	960	2,208,795	43
**2023**	1,418	598	100,656	594	820	2,195,576	37

Table S5a. Unadjusted and adjusted Hazard Ratios (HR) and Confidence Intervals (CI) for RSV-associated ICU admission or death in the full cohort

RSV, Respiratory Syncytial Virus; ICU, Intensive Care Unit; LRTI, Lower respiratory tract Infection.
***Other comorbidities: Life-limiting conditions, oesophageal malformations, cerebral palsy.

	UNADJUSTED HR			ADJUSTED HR		
variable	Hazard Ratio	Lower 95% CI	Upper 95% CI	Hazard Ratio	Lower 95% CI	Upper 95% CI
Male gender	1·10	0·98	1·23	1·10	0·98	1·23
Birth season: summer	0·71	0·58	0·88	0·71	0·58	0·88
Birth season: autumn	1·45	1·22	1·73	1·45	1·22	1·73
Birth season: winter	2·96	2·53	3·46	2·96	2·53	3·46
Small for gestational age	3·65	2·93	4·55	3·91	3·08	4·97
Maternal age 25-29 years	1·11	0·91	1·37	1·30	1·04	1·64
Maternal age 30-34 years	1·35	1·10	1·64	1·57	1·26	1·97
Maternal age ≥ 35 years	1·49	1·21	1·84	1·81	1·43	2·28
Multiple birth	3·44	2·83	4·19	3·43	2·80	4·21
Exposure to smoking in pregnancy	1·22	0·96	1·54	1·27	0·98	1·63
Asthma in the family	1·47	1·29	1·67	1·50	1·31	1·72
One parent born abroad	0·97	0·82	1·14	0·97	0·82	1·14
Both parents born abroad	1·16	1·01	1·33	1·16	1·01	1·33
Parental education: Secondary school	0·90	0·70	1·15	0·90	0·69	1·16
Parental education: University	0·85	0·66	1·08	0·76	0·59	0·99
Having siblings aged 0-3 years	2·90	2·56	3·27	2·92	2·57	3·31
Having siblings aged 4-6 years	1·34	1·15	1·55	1·22	1·05	1·42
Sibling with LRTI before age of 4	2·63	1·72	4·01	2·40	1·54	3·74
Moderate to late preterm	4·54	3·90	5·29	4·86	4·10	5·75
Very preterm	14·15	11·13	18·00	13·93	10·46	18·56
Extremely preterm	31·02	24·36	39·50	33·58	24·62	45·81
Uncomplicated heart disease	5·10	4·02	6·47	4·51	3·41	5·96
Severe Congenital Heart Disease	14·14	10·95	18·26	12·45	9·20	16·85
Chronic lung disease	15·66	12·92	18·99	5·88	4·60	7·52
Down Syndrome	24·28	16·90	34·88	6·57	4·31	10·02
Neonatal respiratory problems	5·80	4·78	7·03	1·54	1·18	2·01
Other comorbidities*	16·04	14·24	18·08	15·46	13·65	17·52

Table S5b. Unadjusted and adjusted Hzard Ratios (HR) and Confidence Intervals (CI) for RSV-associated ICU admission or death in the RSV subpopulation

RSV, Respiratory Syncytial Virus; ICU, Intensive Care Unit; LRTI, Lower respiratory tract Infection.
*****Other comorbidities: Life-limiting conditions, oesophageal malformations, cerebral palsy.

	UNADJUSTED HR			ADJUSTED HR		
variable	Hazard Ratio	Lower 95% CI	Upper 95% CI	Hazard Ratio	Lower 95% CI	Upper 95% CI
Male gender	0·93	0·83	1·04	0·93	0·83	1·04
Birth season: summer	0·63	0·51	0·77	0·63	0·51	0·77
Birth season: autumn	0·71	0·59	0·84	0·71	0·59	0·84
Birth season: winter	1·12	0·96	1·31	1·12	0·96	1·31
Small for gestational age	2·46	1·98	3·07	2·74	2·16	3·47
Maternal age 25-29 years	1·11	0·91	1·37	1·21	0·96	1·51
Maternal age 30-34 years	1·25	1·02	1·52	1·31	1·05	1·65
Maternal age ≥ 35 years	1·35	1·10	1·66	1·47	1·16	1·86
Multiple birth	1·66	1·37	2·02	1·70	1·38	2·08
Exposure to smoking in pregnancy	0·91	0·72	1·15	1·01	0·78	1·30
Asthma in the family	1·01	0·89	1·15	1·05	0·92	1·20
One parent born abroad	1·07	0·90	1·26	1·07	0·90	1·26
Both parents born abroad	1·39	1·20	1·59	1·39	1·20	1·59
Parental education: Secondary school	0·99	0·77	1·27	1·09	0·84	1·41
Parental education: University	1·03	0·81	1·31	1·07	0·83	1·39
Having siblings aged 0-3 years	1·30	1·15	1·47	1·33	1·17	1·50
Having siblings aged 4-6 years	1·13	0·97	1·31	1·06	0·91	1·23
Sibling with LRTI before age of 4	1·20	0·78	1·82	1·18	0·76	1·84
Moderate to late preterm	2·23	1·91	2·60	2·31	1·96	2·74
Very preterm	3·75	2·95	4·77	3·74	2·81	4·98
Extremely preterm	5·18	4·07	6·60	5·70	4·18	7·77
Uncomplicated heart disease	2·42	1·91	3·07	2·32	1·76	3·07
Severe Congenital Heart Disease	3·92	3·04	5·06	4·20	3·13	5·63
Chronic lung disease	3·66	3·02	4·43	1·97	1·53	2·52
Down Syndrome	3·76	2·62	5·40	2·44	1·64	3·63
Neonatal respiratory problems	1·99	1·64	2·42	0·94	0·72	1·21
Other comorbidities*	4·47	3·97	5·04	4·38	3·86	4·96

Table S6a. Sensitivity Analysis - Unadjusted and adjusted Hazard Ratios (HR) and Confidence Intervals (CI) for RSV-associated ICU admission or death in the full cohort including ICD-10 code B97.4

RSV, Respiratory Syncytial Virus; ICU, Intensive Care Unit; LRTI, Lower respiratory tract Infection.
*****Other comorbidities: Life-limiting conditions, oesophageal malformations, cerebral palsy.

	UNADJUSTED HR			ADJUSTED HR		
variable	Hazard Ratio	Lower 95% CI	Upper 95% CI	Hazard Ratio	Lower 95% CI	Upper 95% CI
Male gender	1·11	0·99	1·24	1·11	0·99	1·24
Birth season: summer	0·72	0·59	0·88	0·72	0·59	0·88
Birth season: autumn	1·47	1·23	1·75	1·47	1·23	1·75
Birth season: winter	2·92	2·50	3·41	2·92	2·50	3·41
Small for gestational age	3·68	2·96	4·57	3·91	3·08	4·95
Maternal age 25-29 years	1·11	0·91	1·37	1·31	1·05	1·64
Maternal age 30-34 years	1·35	1·11	1·64	1·59	1·27	1·99
Maternal age ≥ 35 years	1·49	1·21	1·83	1·82	1·44	2·29
Multiple birth	3·45	2·84	4·19	3·44	2·81	4·22
Exposure to smoking in pregnancy	1·19	0·94	1·51	1·24	0·97	1·60
Asthma in the family	1·48	1·30	1·68	1·50	1·31	1·71
One parent born abroad	0·96	0·81	1·14	0·96	0·81	1·14
Both parents born abroad	1·15	1·01	1·33	1·15	1·01	1·33
Parental education: Secondary school	0·90	0·70	1·15	0·90	0·69	1·16
Parental education: University	0·85	0·67	1·08	0·76	0·59	0·98
Having siblings aged 0-3 years	2·87	2·54	3·24	2·88	2·54	3·26
Having siblings aged 4-6 years	1·33	1·15	1·54	1·21	1·04	1·41
Sibling with LRTI before age of 4	2·60	1·71	3·97	2·36	1·51	3·67
Moderate to late preterm	4·55	3·91	5·30	4·88	4·13	5·77
Very preterm	14·10	11·11	17·91	13·94	10·50	18·52
Extremely preterm	30·52	23·96	38·86	33·00	24·20	45·00
Uncomplicated heart disease	5·09	4·02	6·45	4·50	3·42	5·93
Severe Congenital Heart Disease	14·13	10·97	18·21	12·36	9·15	16·69
Chronic lung disease	15·40	12·70	18·67	5·76	4·51	7·36
Down Syndrome	24·67	17·27	35·24	6·75	4·45	10·22
Neonatal respiratory problems	5·70	4·70	6·91	1·52	1·17	1·98
Other comorbidities*	16·11	14·31	18·14	15·56	13·75	17·60

Table S6b. Sensitivity Analysis - Unadjusted and adjusted Hazard Ratios (HR) and Confidence Intervals (CI) for RSV-associated ICU admission or death in the RSV subpopulation including ICD-10 code B97.4

RSV, respiratory Syncytial Virus; LRTI, Lower respiratory tract Infection.
*Other comorbidities: Life-limiting conditions, oesophageal malformations, cerebral palsy.

	UNADJUSTED HR			ADJUSTED HR		
variable	Hazard Ratio	Lower 95% CI	Upper 95% CI	Hazard Ratio	Lower 95% CI	Upper 95% CI
Male gender	0·95	0·85	1·06	0·95	0·85	1·06
Birth season: summer	0·63	0·51	0·77	0·63	0·51	0·77
Birth season: autumn	0·72	0·60	0·86	0·72	0·60	0·86
Birth season: winter	1·12	0·96	1·31	1·12	0·96	1·31
Small for gestational age	2·49	2·00	3·09	2·73	2·16	3·46
Maternal age 25-29 years	1·11	0·91	1·37	1·21	0·97	1·52
Maternal age 30-34 years	1·26	1·04	1·53	1·33	1·07	1·67
Maternal age ≥ 35 years	1·36	1·11	1·67	1·48	1·18	1·87
Multiple birth	1·67	1·38	2·03	1·71	1·39	2·09
Exposure to smoking in pregnancy	0·89	0·70	1·13	0·99	0·77	1·27
Asthma in the family	1·02	0·90	1·16	1·05	0·92	1·20
One parent born abroad	1·07	0·90	1·26	1·07	0·90	1·26
Both parents born abroad	1·37	1·19	1·58	1·37	1·19	1·58
Parental education: Secondary school	0·98	0·77	1·26	1·08	0·83	1·39
Parental education: University	1·04	0·82	1·32	1·07	0·83	1·38
Having siblings aged 0-3 years	1·30	1·15	1·47	1·32	1·17	1·50
Having siblings aged 4-6 years	1·13	0·97	1·30	1·05	0·91	1·23
Sibling with LRTI before age of 4	1·18	0·78	1·80	1·17	0·75	1·83
Moderate to late preterm	2·24	1·92	2·61	2·34	1·98	2·76
Very preterm	3·71	2·92	4·71	3·73	2·81	4·95
Extremely preterm	5·17	4·06	6·58	5·70	4·18	7·78
Uncomplicated heart disease	2·42	1·91	3·07	2·34	1·78	3·09
Severe Congenital Heart Disease	3·93	3·05	5·06	4·20	3·14	5·62
Chronic lung disease	3·60	2·97	4·37	1·92	1·50	2·46
Down Syndrome	3·80	2·66	5·42	2·46	1·66	3·64
Neonatal respiratory problems	1·95	1·61	2·37	0·92	0·71	1·19
Other comorbidities*	4·44	3·94	5·00	4·36	3·86	4·94

Table S7. Sensitivity Analysis - Unadjusted and adjusted Hazard Ratios (HR) and Confidence Intervals (CI) for the variable "Multiple birth" only conducted among full-term multiple births.

ICU, Intensive Care Unit; RSV, Respiratory Syncytial Virus.

	UNADJUSTED HR			ADJUSTED HR		
	**Hazard Ratio**	**Lower 95% CI**	**Upper 95% CI**	**Hazard Ratio**	**Lower 95% CI**	**Upper 95% CI**
**Outcome: Death or ICU admission**						
Full Cohort	2·27	1·61	3·22	2·19	1·52	3·16
RSV Subpopulation	1·30	0·92	1·84	1·30	0·90	1·88
**Outcome: Prolonged Hospitalization**						
Full Cohort	2·60	2·17	3·11	2·53	2·10	3·04
RSV Subpopulation	1·55	1·29	1·85	1·53	1·27	1·84

Table S8. Sensitivity Analysis - Unadjusted and adjusted Hazard Ratios (HR) and Confidence Intervals (CI) for the variable "having a sibling hospitalized for LRTI before the age of 4" only conducted among children with a sibling

ICU, Intensive Care Unit; RSV, Respiratory Syncytial Virus.

	UNADJUSTED HR			ADJUSTED HR		
	**Hazard Ratio**	**Lower 95% CI**	**Upper 95% CI**	**Hazard Ratio**	**Lower 95% CI**	**Upper 95% CI**
**Outcome: Death or ICU admission**						
Full Cohort	2·21	1·50	3·27	2·11	1·41	3·17
RSV Subpopulation	1·29	0·87	1·90	1·30	0·87	1·95
**Outcome: Prolonged Hospitalization**						
Full Cohort	1·37	1·02	1·84	1·38	1·02	1·86
RSV Subpopulation	0·75	0·56	1·01	0·79	0·59	1·06

Table S9a. Unadjusted and adjusted Hazard Ratios (HR) and Confidence Intervals (CI) for RSV-associated prolonged hospitalization in the full cohort

RSV, respiratory Syncytial Virus; LRTI, Lower respiratory tract Infection.
*****Other comorbidities: Life-limiting conditions, oesophageal malformations, cerebral palsy.

	UNADJUSTED HR			ADJUSTED HR		
variable	Hazard Ratio	Lower 95% CI	Upper 95% CI	Hazard Ratio	Lower 95% CI	Upper 95% CI
**Male gender**	1·15	1·08	1·23	1·15	1·08	1·23
**Birth season: summer**	0·76	0·67	0·85	0·76	0·67	0·85
**Birth season: autumn**	1·64	1·48	1·81	1·64	1·48	1·81
**Birth season: winter**	3·13	2·86	3·43	3·13	2·86	3·43
**Small for gestational age**	2·42	2·08	2·82	2·22	1·87	2·62
**Maternal age 25-29 years**	0·97	0·87	1·09	1·06	0·94	1·19
**Maternal age 30-34 years**	1·12	1·01	1·25	1·27	1·13	1·43
**Maternal age ≥ 35 years**	1·25	1·11	1·40	1·40	1·24	1·59
**Multiple birth**	3·69	3·30	4·11	3·63	3·24	4·06
**Exposure to smoking in pregnancy**	1·37	1·22	1·55	1·34	1·18	1·53
**Asthma in the family**	1·29	1·19	1·40	1·24	1·14	1·35
**One parent born abroad**	0·84	0·76	0·93	0·84	0·76	0·93
**Both parents born abroad**	0·97	0·89	1·06	0·97	0·89	1·06
**Parental education: Secondary school**	0·87	0·75	0·99	0·80	0·69	0·92
**Parental education: University**	0·80	0·70	0·92	0·69	0·60	0·80
**Having siblings aged 0-3 years**	2·92	2·72	3·12	2·91	2·71	3·12
**Having siblings aged 4-6 years**	1·30	1·19	1·41	1·22	1·12	1·33
**Sibling with LRTI before age of 4**	1·85	1·38	2·49	1·81	1·34	2·45
**Moderate to late preterm**	3·59	3·27	3·93	3·74	3·38	4·13
**Very preterm**	9·77	8·36	11·42	10·85	9·14	12·87
**Extremely preterm**	16·42	13·68	19·71	19·19	15·46	23·81
**Uncomplicated heart disease**	4·01	3·44	4·67	3·59	3·03	4·25
**Severe Congenital Heart Disease**	9·85	8·31	11·67	7·18	5·85	8·81
**Chronic lung disease**	11·46	10·06	13·05	5·67	4·86	6·61
**Down Syndrome**	19·94	15·98	24·89	7·53	5·84	9·72
**Neonatal respiratory problems**	8·87	8·04	9·79	3·25	2·82	3·75
**Other comorbidities***	9·31	8·63	10·05	9·08	8·39	9·83

Table S9b. Unadjusted and adjusted Hazard Ratios (HR) and Confidence Intervals (CI) for RSV-associated prolonged hospitalization in the RSV subpopulation

RSV, respiratory Syncytial Virus; LRTI, Lower respiratory tract Infection.
*Other comorbidities: Life-limiting conditions, oesophageal malformations, cerebral palsy.

	UNADJUSTED HR			ADJUSTED HR		
variable	Hazard Ratio	Lower 95% CI	Upper 95% CI	Hazard Ratio	Lower 95% CI	Upper 95% CI
Male gender	0·96	0·90	1·03	0·96	0·90	1·03
Birth season: summer	0·63	0·56	0·70	0·63	0·56	0·70
Birth season: autumn	0·76	0·68	0·84	0·76	0·68	0·84
Birth season: winter	1·26	1·15	1·38	1·26	1·15	1·38
Small for gestational age	1·70	1·46	1·97	1·64	1·38	1·94
Maternal age 25-29 years	0·97	0·87	1·09	0·99	0·87	1·11
Maternal age 30-34 years	1·06	0·96	1·18	1·09	0·97	1·23
Maternal age ≥ 35 years	1·16	1·04	1·30	1·18	1·04	1·34
Multiple birth	1·88	1·68	2·09	1·87	1·67	2·09
Exposure to smoking in pregnancy	1·01	0·89	1·13	1·05	0·92	1·19
Asthma in the family	0·88	0·81	0·96	0·86	0·79	0·94
One parent born abroad	0·94	0·85	1·04	0·94	0·85	1·04
Both parents born abroad	1·11	1·02	1·21	1·11	1·02	1·21
Parental education: Secondary school	0·95	0·83	1·10	0·96	0·83	1·11
Parental education: University	0·99	0·86	1·13	0·96	0·83	1·11
Having siblings aged 0-3 years	1·33	1·24	1·42	1·33	1·24	1·43
Having siblings aged 4-6 years	1·08	1·00	1·18	1·04	0·96	1·14
Sibling with LRTI before age of 4	0·80	0·59	1·08	0·84	0·62	1·14
Moderate to late preterm	1·83	1·67	2·00	1·83	1·66	2·03
Very preterm	2·71	2·31	3·16	2·93	2·47	3·48
Extremely preterm	2·94	2·45	3·53	3·40	2·74	4·21
Uncomplicated heart disease	2·01	1·73	2·35	1·95	1·64	2·31
Severe Congenital Heart Disease	2·88	2·43	3·41	2·61	2·14	3·18
Chronic lung disease	2·79	2·45	3·18	1·97	1·69	2·30
Down Syndrome	3·56	2·85	4·45	2·64	2·08	3·36
Neonatal respiratory problems	3·08	2·79	3·41	1·91	1·66	2·20
Other comorbidities*	3·03	2·81	3·27	3·03	2·80	3·28

Table S10a. Sensitivity Analysis - Unadjusted and adjusted Hazard Ratios (HR) and Confidence Intervals (CI) for RSV-associated prolonged hospitalization in the full cohort including ICD-10 code B97.4

RSV, Respiratory Syncytial Virus; ICU, Intensive Care Unit; LRTI, Lower respiratory tract Infection.
***Other comorbidities: Life-limiting conditions, oesophageal malformations, cerebral palsy.

	UNADJUSTED HR			ADJUSTED HR		
variable	Hazard Ratio	Lower 95% CI	Upper 95% CI	Hazard Ratio	Lower 95% CI	Upper 95% CI
Male gender	1·15	1·08	1·23	1·15	1·08	1·23
Birth season: summer	0·76	0·68	0·86	0·76	0·68	0·86
Birth season: autumn	1·64	1·49	1·82	1·64	1·49	1·82
Birth season: winter	3·11	2·84	3·40	3·11	2·84	3·40
Small for gestational age	2·44	2·10	2·83	2·30	1·94	2·72
Maternal age 25-29 years	0·97	0·87	1·09	1·06	0·94	1·20
Maternal age 30-34 years	1·12	1·01	1·25	1·27	1·13	1·43
Maternal age ≥ 35 years	1·25	1·12	1·40	1·41	1·25	1·60
Multiple birth	3·65	3·28	4·08	1·38	1·22	1·56
Exposure to smoking in pregnancy	1·40	1·25	1·58	1·38	1·22	1·56
Asthma in the family	1·28	1·18	1·38	1·23	1·13	1·34
One parent born abroad	0·86	0·78	0·94	0·86	0·78	0·94
Both parents born abroad	0·97	0·89	1·06	0·97	0·89	1·06
Parental education: Secondary school	0·87	0·75	0·99	0·82	0·71	0·95
Parental education: University	0·80	0·70	0·91	0·71	0·61	0·82
Having siblings aged 0-3 years	2·88	2·69	3·09	2·90	2·70	3·10
Having siblings aged 4-6 years	1·29	1·19	1·40	1·21	1·11	1·31
Sibling with LRTI before age of 4	1·99	1·49	2·64	1·95	1·46	2·60
Moderate to late preterm	3·61	3·29	3·96	3·77	3·41	4·18
Very preterm	9·87	8·46	11·51	10·34	8·64	12·37
Extremely preterm	16·74	13·99	20·04	19·03	15·23	23·77
Uncomplicated heart disease	3·96	3·40	4·62	3·44	2·88	4·11
Severe Congenital Heart Disease	9·76	8·24	11·56	7·02	5·69	8·67
Chronic lung disease	11·77	10·36	13·38	5·97	5·12	6·96
Down Syndrome	20·12	16·17	25·04	7·66	5·96	9·85
Neonatal respiratory problems	9·00	8·17	9·92	3·36	2·91	3·88
Other comorbidities*	9·53	8·84	10·27	9·29	8·59	10·05

Table S10b. Sensitivity Analysis - Unadjusted and adjusted Hazard Ratios (HR) and Confidence Intervals (CI) for RSV-associated prolonged hospitalization in the RSV subpopulation including ICD-10 code B97.4

RSV, Respiratory Syncytial Virus; ICU, Intensive Care Unit; LRTI, Lower respiratory tract Infection.
***Other comorbidities: Life-limiting conditions, oesophageal malformations, cerebral palsy.

	UNADJUSTED HR			ADJUSTED HR		
variable	Hazard Ratio	Lower 95% CI	Upper 95% CI	Hazard Ratio	Lower 95% CI	Upper 95% CI
**Male gender**	0·97	0·91	1·03	0·97	0·91	1·03
**Birth season: summer**	0·62	0·55	0·70	0·62	0·55	0·70
**Birth season: autumn**	0·76	0·69	0·84	0·76	0·69	0·84
**Birth season: winter**	1·26	1·15	1·38	1·26	1·15	1·38
**Small for gestational age**	1·71	1·47	1·98	1·68	1·42	1·99
**Maternal age 25-29 years**	0·98	0·87	1·09	0·99	0·88	1·11
**Maternal age 30-34 years**	1·07	0·96	1·19	1·10	0·97	1·23
**Maternal age ≥ 35 years**	1·18	1·05	1·32	1·20	1·06	1·35
**Multiple birth**	1·86	1·67	2·08	1·86	1·66	2·08
**Exposure to smoking in pregnancy**	1·03	0·92	1·16	1·08	0·95	1·22
**Asthma in the family**	0·87	0·80	0·94	0·85	0·78	0·93
**One parent born abroad**	0·95	0·86	1·05	0·95	0·86	1·05
**Both parents born abroad**	1·11	1·02	1·21	1·11	1·02	1·21
**Parental education: Secondary school**	0·95	0·83	1·10	0·99	0·86	1·14
**Parental education: University**	0·99	0·86	1·13	0·99	0·86	1·14
**Having siblings aged 0-3 years**	1·33	1·24	1·42	1·33	1·24	1·43
**Having siblings aged 4-6 years**	1·08	0·99	1·18	1·04	0·95	1·13
**Sibling with LRTI before age of 4**	0·86	0·65	1·15	0·91	0·68	1·22
**Moderate to late preterm**	1·84	1·68	2·02	1·87	1·69	2·06
**Very preterm**	2·71	2·32	3·16	2·76	2·31	3·31
**Extremely preterm**	3·04	2·54	3·63	3·40	2·72	4·25
**Uncomplicated heart disease**	2·00	1·72	2·33	1·87	1·57	2·23
**Severe Congenital Heart Disease**	2·85	2·41	3·38	2·62	2·13	3·21
**Chronic lung disease**	2·87	2·53	3·27	2·06	1·76	2·40
**Down Syndrome**	3·56	2·86	4·43	2·65	2·09	3·36
**Neonatal respiratory problems**	3·12	2·83	3·44	1·98	1·72	2·29
**Other comorbidities***	3·06	2·84	3·30	3·06	2·83	3·31

Table S11. Prevalence of underlying comorbidities by outcome group

Data are reported as numbers (N) with percentages· Values <5 are reported as "<5," and corresponding percentages are not provided.
ICU, Intensive Care Unit; WHO, World Health Organization.
***Other comorbidities: Life-limiting conditions, oesophageal malformations, cerebral palsy.

	Death N(%)	ICU admission N(%)	Prolonged hospitalization N(%)
**Total sample**	27 (100·0)	1,210 (100)	3,766 (100·0)
**Classification of prematurity according to WHO**			
Full term	20 (74·1)	868 (71·8)	2,901 (77·2)
Moderate to late preterm	5 (18·5)	200 (16·5)	552 (14·7)
Very preterm	0 (0·0)	72 (6·0)	186 (4·9)
Extremely preterm	<5	69 (5·7)	121 (3·2)
**Atrial Septal Defect or Ventricular Septal Defect diagnosis**	<5	71 (5·9)	175 (4·6)
**Severe congenital heart defect diagnosis**	<5	62 (5·1)	140 (3·7)
**Down syndrome diagnosis**	0 (0·0)	30 (2·5)	80 (2·1)
**Any chronic lung disease diagnosis**	<5	111 (9·2)	245 (6·5)
**Diagnosis of neonatal respiratory problems of the term child**	5 (18·5)	112 (9·3)	468 (12·4)
**Other comorbidities***	18 (66·7)	391 (32·3)	878 (23·3)
